# Complex Energy Landscapes of Self-Assembled Vesicles

**DOI:** 10.1021/jacs.3c04285

**Published:** 2023-07-10

**Authors:** Jiabin Luan, Danni Wang, Shaohua Zhang, Yusuke Miyazaki, Wataru Shinoda, Daniela A. Wilson

**Affiliations:** † Institute for Molecules and Materials, Radboud University Nijmegen, Heyendaalseweg 135, 6525 AJ Nijmegen, The Netherlands; ‡ Research Institute for Interdisciplinary Science, Okayama University, Okayama 700-8530, Japan

## Abstract

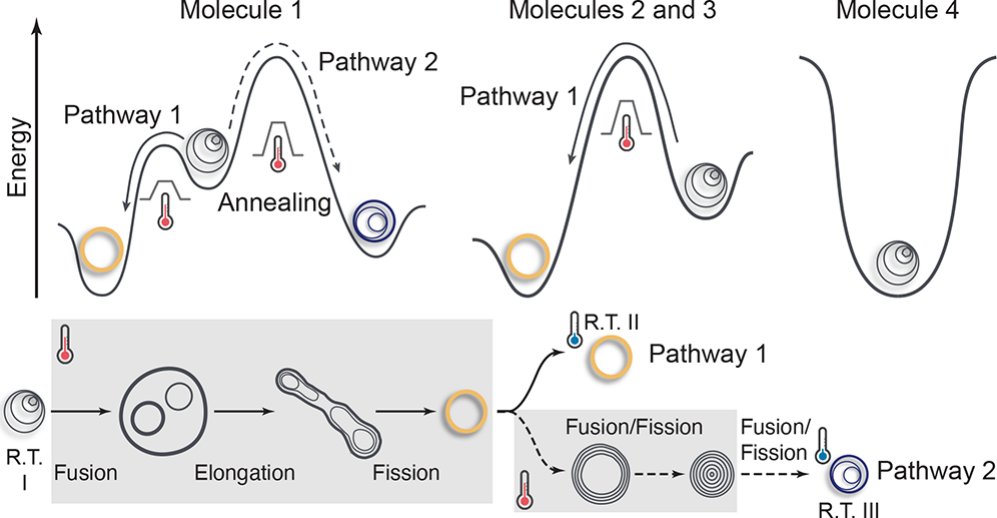

The field of supramolecular chemistry has witnessed tremendous progress in bringing the system away from equilibrium for traditionally inaccessible structures and functions. Vesicular assemblies with complex energy landscapes and pathways, which are reminiscent of diverse cellular vesicles like exosomes, remain exceedingly rare. Here, relying on the activation of oligo(ethylene glycol) (OEG) interdigitation and the encoded conformational freedom in monodisperse Janus dendrimers, we reveal a rich landscape and a pathway selection of distinct vesicles. The interdigitation can be selectively switched on and off using temperature ramps, and the critical temperatures can be further determined by molecular design. Our findings suggest that synthetic vesicles, with different energy states and unexpected transition pathways, emulate dynamic cellular vesicles in nature. We anticipate that vesicles with an activated OEG corona conformation will open new routes for nanomedicine and advanced materials.

## 1 Introduction

Supramolecular assemblies are ubiquitous in nature and fundamental to the formation of essential units for complex biological functions. Compelling illustrations are the actin polymerization of the cytoskeleton and the lipid bilayers of the cellular membrane, among many others. Resonating with the living systems, supramolecular chemistry^1^ fascinates with the rich functionality it creates from hierarchically self-assembled architectures. The traditional premise in self-assembly relies on the molecular design of building blocks to assemble desired structures under thermodynamic equilibrium and further to direct their potential functionality.^2^ Under thermodynamic control, the morphology of self-assembled molecules consistently remains in the deepest energy well unless alterations are made to the composition and/or temperature of the system.^2^ Nature, on the other hand, adopts a cannier strategy by bringing the biomolecular systems out of equilibrium to achieve rich energy landscapes with distinct functions, therefore precluding the needs for the evolution of new molecules. This renders unique features such as adaptivity and diversity to the biomolecular systems. Exosomes, for example, display great morphological variability, including unilamellar vesicles (ULVs, single bilayer vesicles) and multivesicular vesicles (MVVs, also known as vesosomes) from a single cell type, which were believed to have different biological functions in cellular communication.^3^ Under kinetic control, the history of the self-assembled molecules significantly influences their structures prior to, during, or following the preparation process. These kinetically trapped states of assemblies in local energy minima, along with their thermodynamic state, compose the energy landscape with diverse structures and potentially unexpected functions.^2,4–7^

By adopting this novel strategy, it has recently been shown the possibility to use supramolecular polymers^8^ to navigate across intricate energy landscapes of mostly one-dimensional assemblies with multiple local energy wells en route to the thermodynamically favored state.^4,5,9,10^ Notably, unique pathway selection during preparation was encoded in the design of the molecular building blocks to direct the structure and functions of the system.^11–14^ Rational designing rules based on kinetic pathway guidance to establish well-defined energy landscapes are always nontrivial and difficult to devise. Substantial progress in supramolecular chemistry, as exemplified by one-dimensional fibers^11^ and two-dimensional sheets,^15^ illustrates that antagonistic intermolecular interactions are mechanistically essential.^16^ However, progress toward the complex landscape has not been translated to compartmentalized three-dimensional vesicles. The gap stems from the fact that, comparing with the well-known supramolecular fiber systems, which are rich of competing interactions, such as hydrogen bonding,^11,13,15^ it is challenging to switching on and off the dominant van der Waals interaction (hydrophobic effects) within the hydrophobic region of the membrane of vesicles. This presents a significant obstacle to navigating distinct vesicles within the same energy landscape. Recently, our group showed that the classical hydrophilic poly(ethylene glycol) could be activated, resulting in programmable surface functionalization of vesicles.^17^ Moreover, the formation of self-assembled monolayers could be driven by dipole–dipole interdigitation of oligo(ethylene glycol) (OEG) chains, although in the organic solvent of toluene.^18^ We hypothesized that if the interdigitation of the OEG chains in the hydrophilic region could be switched on or off in competition with the overall molecular conformational freedom, it should be possible to bring the vesicles out-of-equilibrium, resulting in a rich energy landscape.

**Figure 1:**
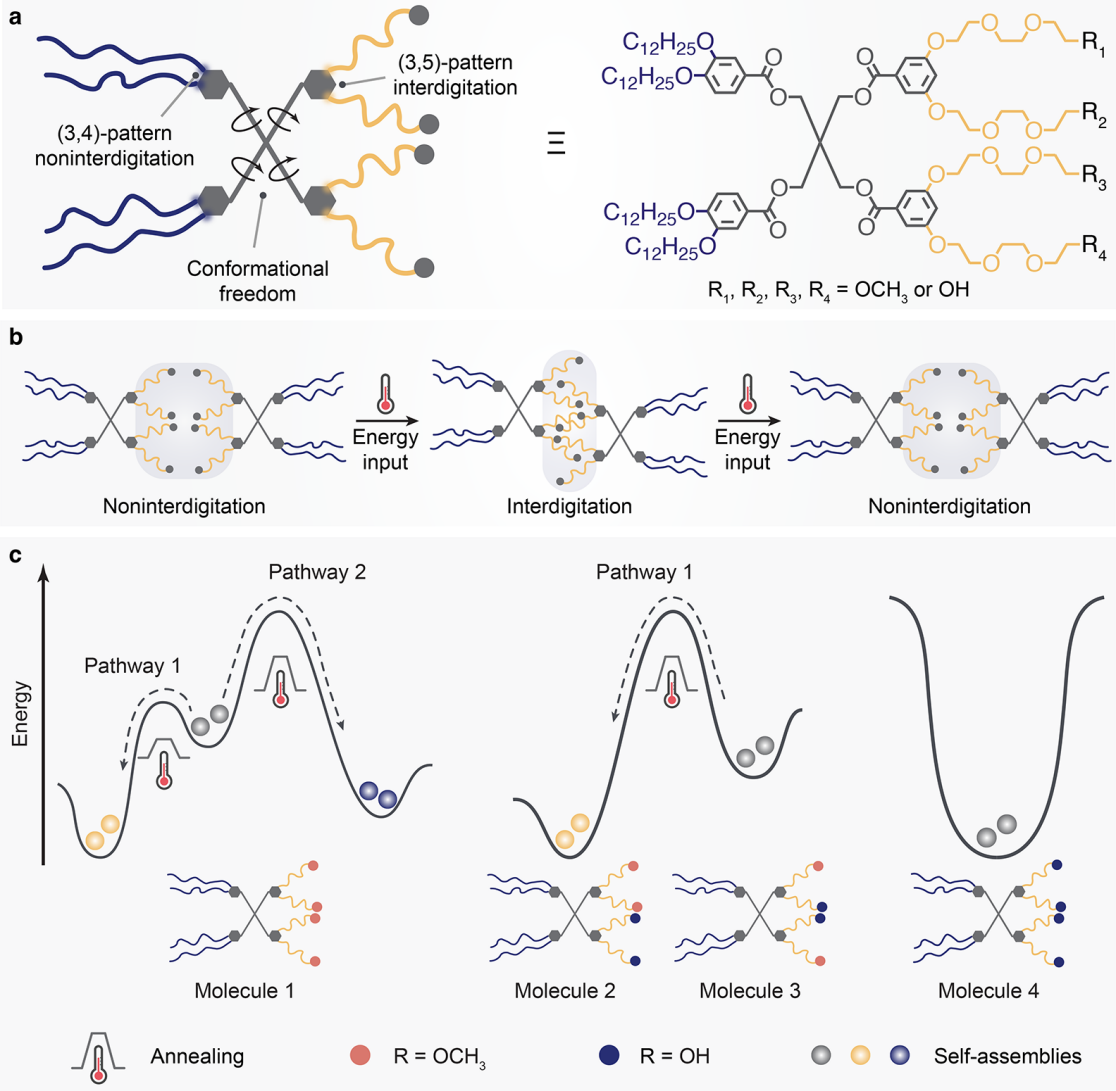
Energy landscapes and pathway selection of Janus dendrimer self-assemblies. (a) Design of molecular building blocks. The molecules of Janus dendrimers consist of hydrophilic OEG with different end groups (–OCH_3_ or –OH) and hydrophobic dodecyloxy chains on the phenolic acid units. These two amphiphilic blocks are connected by a pentaerythritol core. (b) Schematic representation of the temperature-induced interdigitation and noninterdigitation of OEG chains from Janus dendrimer molecules. (c) Free energy landscapes of Janus dendrimer assemblies. Different end groups of the OEG blocks are designed to modulate the competing interactions, which results in distinct pathway selections.

To test our hypothesis, we use self-assembled vesicles from Janus dendrimer molecules, which offer both high stability and flexibility.^19,20^ At the core of our approach is the critical discovery that traditionally inert OEG chains undergo interdigitation during heating, which can be further disrupted due to the increased conformational freedom of molecules. In the seminal work by Percec and co-workers, the synthetic versatility and precise control over molecular structure and functionality offer Janus dendrimers unique attributes to compete with lipids, polymers, or surfactants.^19^ In this work, we investigated Janus dendrimers with (3,5)-patterned hydrophilic OEG and (3,4)-patterned hydrophobic aliphatic chains on phenolic acid units connected by a pentaerythritol core. Such molecular design has the following merits (Figure 1a): (1) the (3,5)-pattern provides conformational probability^21^ for the interdigitation of OEG chains, which has never been realized in the aqueous environment; (2) the (3,4)-pattern of hydrophobic aliphatic blocks with prominent steric effect, on the contrary, hinders their internal interdigitation, which offers the membrane more flexibility; (3) the pentaerythritol core, with four carbon–carbon single bonds capable of rotating/flipping,^22^ provides extra conformational freedom for the essential competing interaction to disrupt the potential OEG interdigitation. Because both the dipole–dipole interdigitation and the conformational freedom of molecules are dependent on temperature, we envision that temperature can be applied as a model parameter to guide vesicles to different energy states within the landscape (Figure 1b). Furthermore, to generalize our finding, we also encoded the critical temperature, which defines the balance between the competing interactions, in the design of the molecules, resulting in a pathway selection between distinct structures at a body temperature of 37 °C (Figure 1c).

## 2 Results and Discussion

### 2.1 Energy Landscape in the Presence of Ethanol

**Figure 2:**
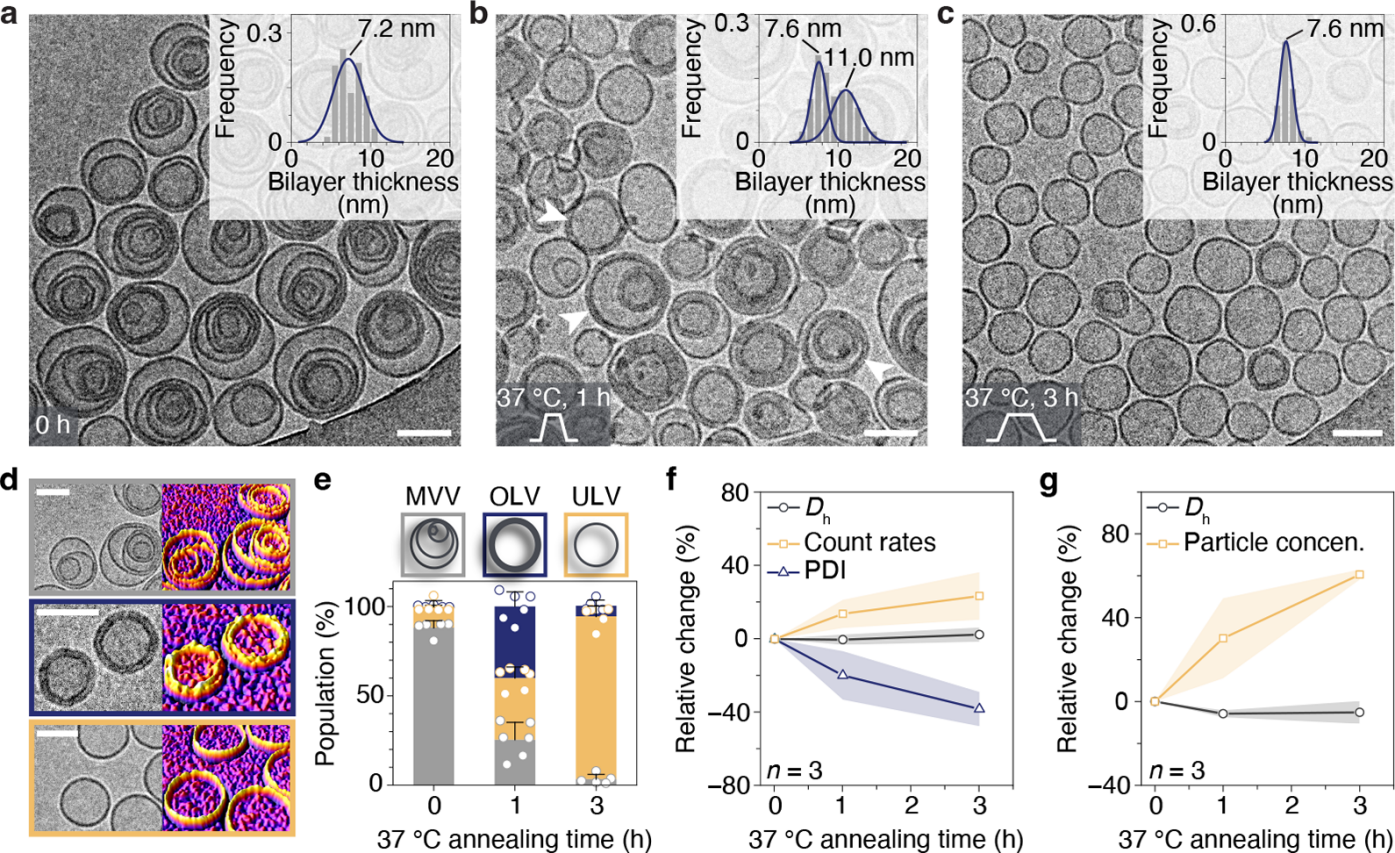
Assessment of the morphological transition of self-assembled Janus dendrimers via 37 °C annealing in the presence of ethanol. (a–c) Cryo-TEM images of self-assemblies prepared by direct injection (a) and assemblies annealed from 37 °C after being equilibrated for 1 (b) and 3 h (c). Vesicles with two stacked bilayers are highlighted by arrows in (b). The insets display frequency histograms illustrating the distribution of the bilayer thickness. Each histogram was fitted with the Gaussian distribution, as depicted by the solid lines. (d) Typical morphologies of self-assemblies at different energy states, as indicated by different color codes [as used in the bar graphs in (e)]: eccentric MVVs (gray), OLVs (blue), and ULVs (yellow). 3D surface plots were generated by ImageJ, and details can be found in Experimental Section. Scale bars are 100 nm. (e) Quantitative measurement of assemblies obtained from direct injection (a) and post-treatment at 37 °C (b,c). For each condition, images from different areas and different batches of samples were taken and counted to minimize the error (*n* > 500 particles). See Supporting Information for a detailed description (Figure S5). (f,g) Relative change of hydrodynamic diameter *D*_h_ (%), derived count rates (%), PDI (%) as measured by DLS (f) and *D*_h_ (%), and particle concentration (%) as measured by NTA (g) as a function of annealing time at 37 °C. Characterization was performed on three different batches of samples (*n* = 3).

The formation of dendrimersomes was induced by the injection of the ethanol solution of Janus dendrimer (3,4)12G1-PE-(3,5)-3EO-G1-(OCH_3_)_4_ (Scheme S1 and Figure S1) into Milli-Q water. We first investigated the energy landscape of the system in the presence of ethanol, which is a good solvent of dendrimer. Instead of the thermodynamically favorable ULVs, we obtained mostly nonconcentric MVVs with a percentage of nearly 90% as quantified by cryogenic transmission electron microscopy (cryo-TEM) images (Figure 2a,e, and see Figure S2 for details of various kinds of vesicles). Such eccentric vesicles were shown to reside in a relatively deep energy well because the formation of MVVs was insensitive to variants in the preparation and characterization procedures. Specifically, neither the centrifugation postassembly nor parameters during assembly such as dissolving time and concentration of dendrimer, solvent, or injection speed would alter the structure and composition of MVVs (Figure S3).

After equilibrating an as-assembled solution from temperature such as the body temperature of 37 °C for 1–3 h followed by annealing, MVVs gradually show a transition to a different energy landscape of ULV morphology (Figure 2b,c). A mixture of MVVs, ULVs, and an intermediate type of oligolamellar vesicles (OLVs, mostly two-bilayer vesicles) was present if solution was annealed from 37 °C for 1 h (Figure 2b,d). During this transition, of particular note is the appearance of OLVs and some of the MVVs, showing “thicker” membranes of vesicles (arrows in Figure 2b). The thickness of the “thicker” membrane is mostly around 14.5 nm, which corresponds to two bilayers stacked together, which provides clear evidence of fusion during the 1 h annealing process. As longer equilibration time was allowed, fission of vesicles took place, leading to the production of over 90% of ULVs (Figure 2c,e). By mapping the distribution of the bilayer thickness, we could see a clear transition in the bilayer thickness during the annealing process, indicating the occurrence of fusion and then fission (insets in Figure 2a–c). Further extending the annealing treatment at 37 °C from 6 to 23 h did not result in significant change of population of vesicles, with ULVs being the dominant species (Figure S4). Dynamic light scattering (DLS) and nanoparticle tracking analysis (NTA) were further used to monitor the possible overall change of the two different states after annealing. We found an increase in concentration after annealing, as reflected by the increased count rates in DLS and particle concentration in NTA, respectively (Figure 2f,g). On the other hand, the size of samples remained surprisingly almost the same after the annealing treatment (see Supporting Information for discussion). DLS further revealed a decrease in PDI, suggesting that the transformation to ULVs generates more monodispersed vesicles. Taken together, ULVs are the thermodynamically favored product in the presence of ethanol and thus correspond to the deepest well in this energy landscape. Through a fusion–fission process, internal vesicles of MVVs are brought outside into direct contact with the bulk solution via the formation of ULVs. This transition of vesicles further enhances the monodispersity of assemblies.

### 2.2 Energy Landscape in the Absence of Ethanol

**Figure 3:**
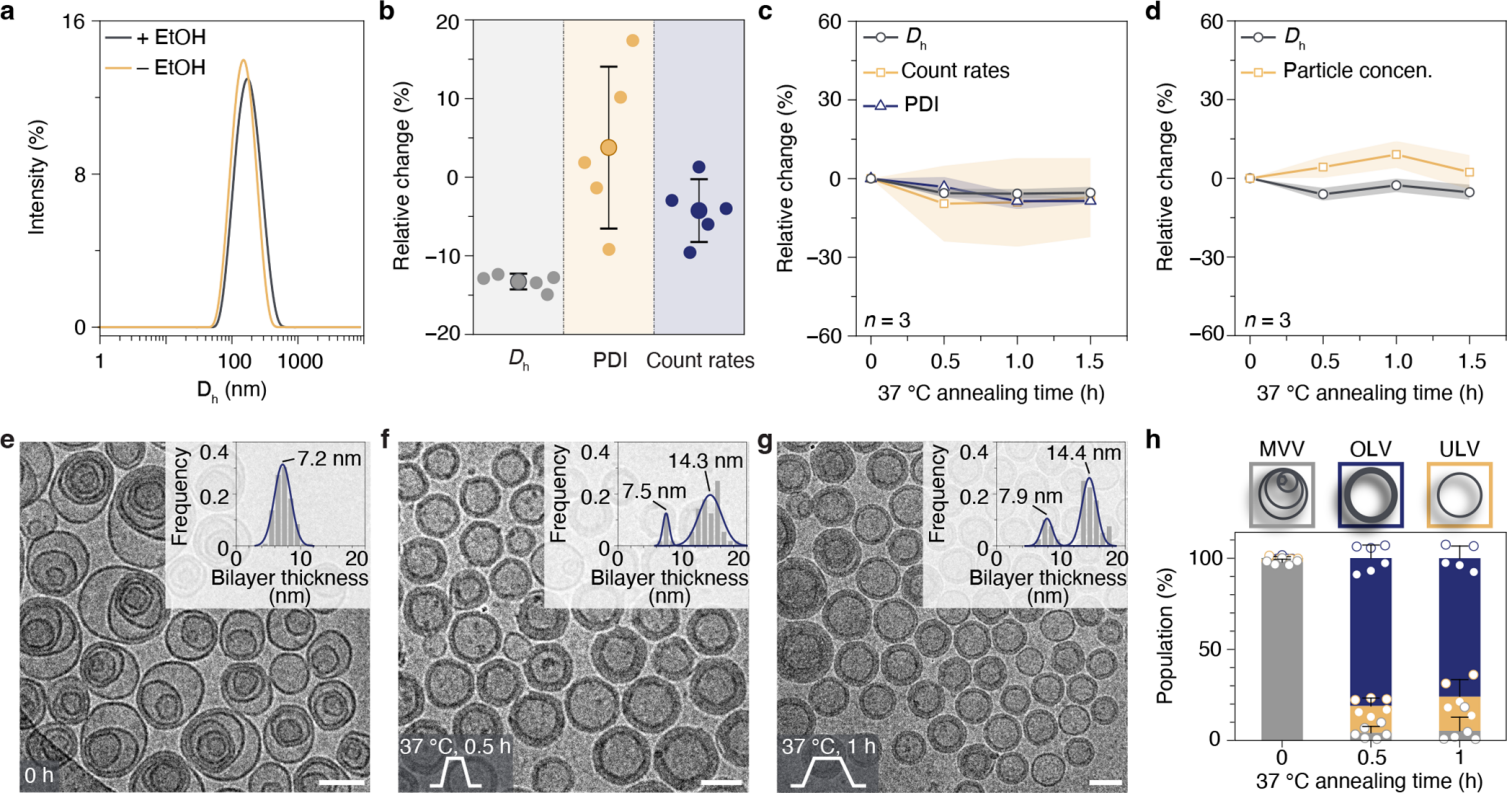
Assessment of the morphological transition of self-assembled Janus dendrimers via 37 °C annealing in the absence of ethanol. (a) *D*_h_ profiles as determined by DLS in the presence (+EtOH) and absence (−EtOH) of ethanol. (b) Relative change of *D*_h_ (%), PDI (%), and derived count rates (%) as compared with the sample with ethanol as measured by DLS. (c,d) Relative change of *D*_h_ (%), derived count rates (%), PDI (%) as measured by DLS (c), and *D*_h_ (%) and particle concentration (%) as measured by NTA (d) of the sample without ethanol as a function of annealing time at 37 °C. Characterization was performed on three different batches of samples (*n* = 3). (e–g) Cryo-TEM images of self-assemblies after removing EtOH (e) and assemblies annealed from 37 °C after being equilibrated for 0.5 (f) and 1 h (g). The insets display frequency histograms illustrating the distribution of the bilayer thickness. Each histogram was fitted with the Gaussian distribution, as depicted by the solid lines. (h) Quantitative measurement of assemblies obtained from direct injection (e) and post-treatment at 37 °C (f,g). For each condition, images from different areas and different batches of samples were taken and counted to minimize the error (*n* > 500 particles).

Ethanol has been shown to induce interdigitation of lipid alkyl chains, resulting in the fusion of vesicles.^23^ To understand the role of the solvent in the two energy states, we then studied the energy landscape in a medium without ethanol. The same injection method was performed to induce self-assembly, followed by the removal of ethanol by thorough dialysis with frequent changes of the medium of Milli-Q water. We first investigated the effect of ethanol removal on the size and distribution of assemblies. There were no significant changes in PDI and count rates, while a slight decrease from 160 to 150 nm in size was observed (Figure 3a,b). It is known that ethanol causes an increase in the area per lipid molecule, which leads to a subsequent increase in the size of the assembly.^24^ This might also be applicable in our case, as the Janus dendrimer bears similar hydrophobic alkyl chains as lipids. Nevertheless, the decrease in size is not significantly noticeable, and assemblies show the same monodisperse peak in DLS. This was further substantiated by the same nonconcentric MVVs (>90% over 500 particles counting) with a similar distribution of bilayer thickness as that before removing ethanol in cryo-TEM studies (Figure 3e,h). The removal of ethanol by dialysis had a limited effect, and the vesicles remained in the same energy state. We next applied the same annealing procedure to monitor the relative changes in size and concentration after thermal treatment. Different from the results in the presence of ethanol, no significant difference in particle size and concentration after the annealing treatment up to 1.5 h was detected as characterized by DLS and NTA (Figure 3c,d). This seems to indicate that the assemblies remain in the same energy state regardless of 37 °C annealing in the absence of ethanol. However, both DLS and NTA could not provide a definite answer to the above assumption before examining the morphology of the assemblies. Unexpectedly, after the annealing treatment for half an hour, cryo-TEM revealed a new type of OLVs with a population of over 80% (Figure 3f,h). By plotting the profile of bilayer thickness, most of the OLVs were constituted by two stacked bilayers, as the peak of the Gaussian distribution located around 14.3 nm, which is almost twice as much as the single bilayer thickness (inset in Figure 3f). Further extension of the annealing time at 37 °C from 1 to 48 h did not result in significant changes of the composition of different structures (Figures 3g and S6). The above observations show that the absence of ethanol shifts the final state of MLVs from ULVs to OLVs after the annealing experiments. Ethanol, as a good solvent for the dendrimer, therefore serves as a modulator to achieve different vesicular states. Such modulating role of solvent has also been observed in other supramolecular systems.^5^

### 2.3 Energy Landscapes Modulated by Temperature

**Figure 4:**
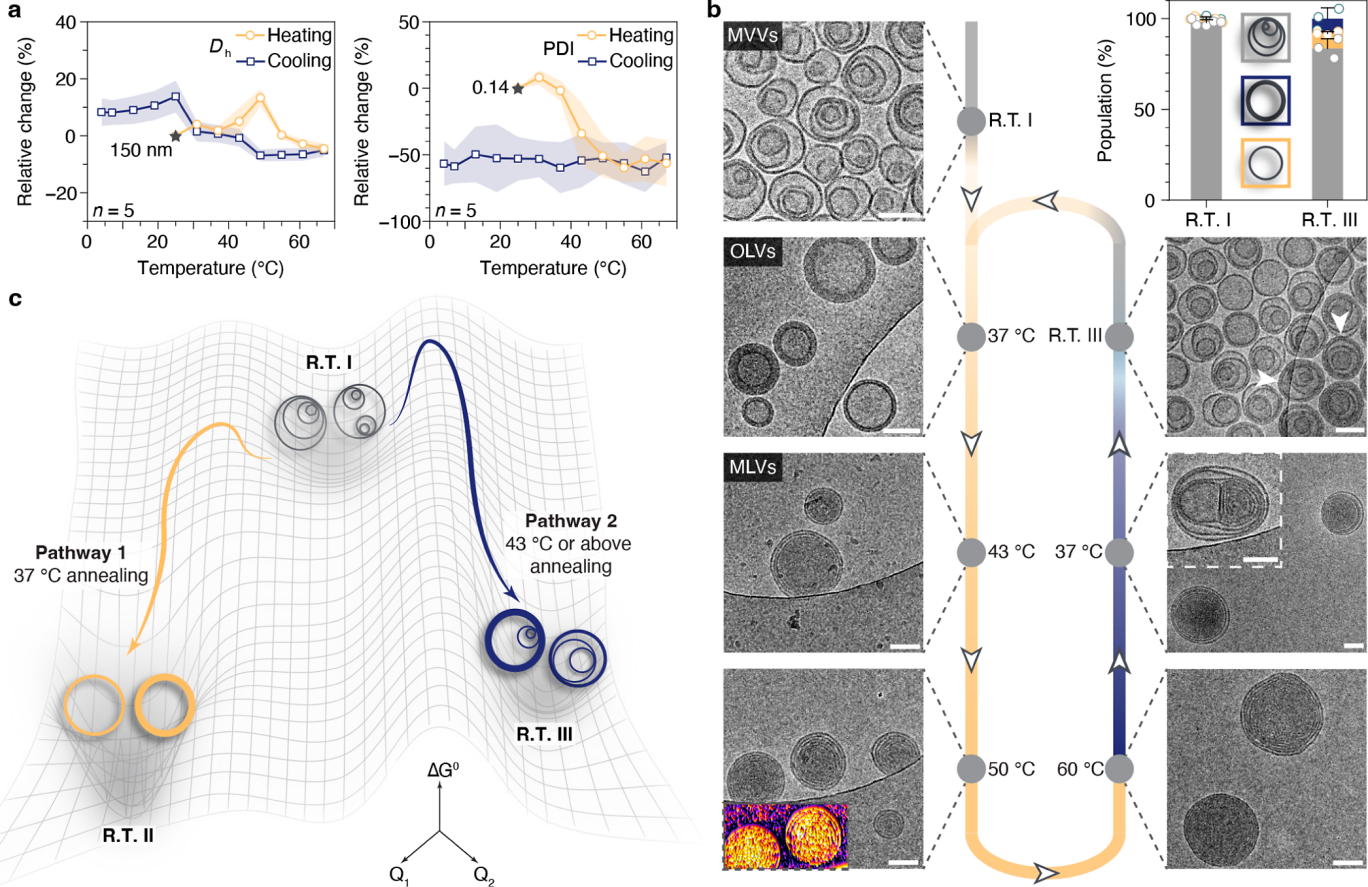
Energy landscapes and pathway selection of assemblies navigated by temperatures. (a) Relative change of *D*_h_ (%) and PDI (%) of assemblies as monitored by the temperature trend measurements in DLS. Characterization was performed on five different batches of samples (*n* = 5). (b) Cryo-TEM images of self-assemblies as vitrified at the indicated temperatures during the first heating/cooling cycle. Scale bars are 100 nm. Vesicles with two stacked bilayers are highlighted by arrows in R.T. III. (c) Schematic illustration of the pathway selection of assemblies at R.T. as modulated by the annealing temperature.

The annealing treatment at 37 °C has been demonstrated to be a key indicator in guiding the self-assembly pathways of dendrimersomes. We next carried out a closer inspection of the effect of temperature. To avoid any interference of the solvent, all of the following experiments were performed with samples without ethanol. The change in size and PDI of the sample relative to the starting point at 25 °C was monitored in heating and cooling cycles (Figure 4a). Samples were stable in all testing temperatures with a monodisperse distribution. Starting at around 150 nm in hydrodynamic diameter, the sample exhibited a limited fluctuation between 150 and 170 nm in the temperature range of 4–67 °C, while for PDI, a more noticeable change was observed between 37 and 43 °C with a reduced PDI of 0.07, approximately half of the initial value of 0.14. This significant drop in PDI indicates that the sample becomes narrower in the dimensional distribution. Adjusting the heating and cooling rate during the temperature trend as well as successive heating and cooling cycles did not bring significant differences (Figure S7).

To confirm the effect of temperature on the morphologies of dendrimersomes, we followed the structures by cryo-TEM during the whole annealing procedure (Figure 4b). High-quality cryo-TEM images can be acquired at or below a room temperature (R.T.) of 23 °C. However, it is rather less frequent and challenging to capture structures above the R.T. due to a higher speed of water evaporation, which could cause problems in obtaining grids with ideal ice thickness. We adapted a recently developed method^25^ and successfully preserved vesicular structures at temperatures as high as 60 °C, which is the highest temperature that could be obtained with the Vitrobot vitrification system (see Experimental Section for detailed description and parameters). The initial MVVs remained unchanged at 7 °C after a cooling process (Figure S8). Heating the self-assembled solutions to the previously annealed temperature of 37 °C unambiguously showed a transition from MVVs to OLVs. Together with the result in Figure 3f, it showed that assemblies shared the same state of OLVs at 37 °C and R.T. after annealing from 37 °C. Continuing the heating process to 43 °C would shift the OLVs uniformly to a new state of multilamellar vesicles (MLVs, also known as onion vesicles). The increased temperature provided extra energy to the system to generate more uniform MLVs, as evidenced by the sharp decrease in PDI (Figure 4a). Further energy supply by heating to 50 and 60 °C did not transform the MLVs anymore. These transitions in different morphologies were not affected by the adjustment of the heating speed (Figure S9). We then followed the assemblies in the cooling process. After cooling to 37 °C for half an hour, the majority of vesicles remained MLVs with infrequent encounter of OLVs and MVVs. The captured snapshot of the intermediate vesicle indicated that fission takes place during the transition between different states (inset of 37 °C in cooling, Figure 4b). A much more dramatic change in morphologies could be detected after cooling the sample to R.T. with more than 80% of MVVs again being formed. We denote samples after initial self-assembly, annealing from 37 °C and annealing from above 43 °C as R.T. I, R.T. II, and R.T. III, respectively. This is to distinguish the three different states of vesicles at the same temperature of R.T. Although the largest populations of samples R.T. I and R.T. III are both MVVs, the annealed sample from above 43 °C (R.T. III) is not exactly the same as the initial sample (R.T. I), as demonstrated by larger populations of ULVs and OLVs (the bar chart in Figure 4b). Moreover, the MVVs at two states were also different, with quite a lot of MVVs showing several stacked bilayers, as with the appearance of thicker membranes in R.T. III (arrows in R.T. III, Figure 4b). Varying the cooling speed from 43 °C or above 43 °C to R.T. did not result in any difference in the transition (Figure S10). The indication of a different energy state of vesicles of R.T. III motivated us to examine a second cycle of heating and cooling. It turned out that the transitions observed in the first cycle were also reproducible in the second cycle (Figure S11). Together with the results in the previous section (Figure 3f–h), a critical annealing temperature at 37 or 43 °C was revealed to control the pathway selectivity of uniform OLVs and MVVs at R.T., respectively (Figure 4c). These findings demonstrate the unique temperature-driven energy landscapes of vesicles and highlight that the thermal history of vesicles will determine the position of the final product in the energy landscape.

**Figure 5:**
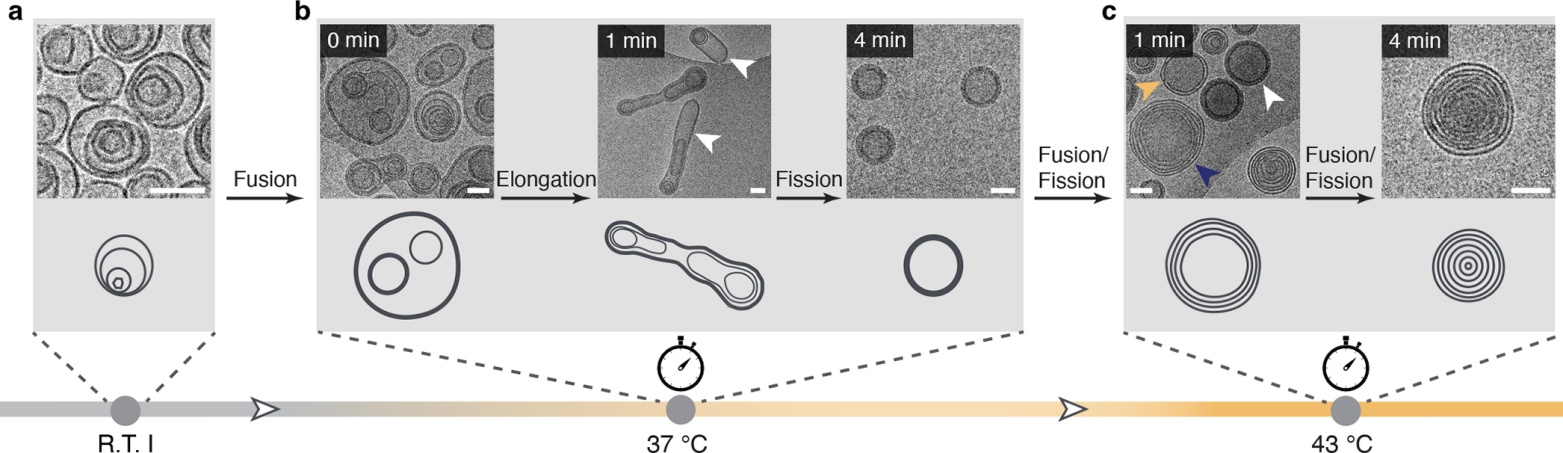
Assessment of the morphological transition mechanism at different temperatures. Snapshots of cryo-TEM images of self-assemblies: (a) at room temperature (R.T. I), (b) 0, 1, and 4 min at 37 °C, and (c) 1 and 4 min at 43 °C. Scale bars are 100 nm. Tubular vesicles with multiple stacked bilayers are highlighted by arrows in (b) for 1 min. Initial OLVs with stacked bilayers, OLVs with discernible bilayers, and immature MLVs are highlighted by white, yellow, and blue arrows in (c) 1 min, respectively.

To investigate the mechanism behind these remarkable transitions between morphologies, we set out to visualize the evolution of self-assemblies in real-time by cryo-TEM (Figures 5 and S12). We plunged samples into liquid ethane to rapidly vitrify the sample within 0 to 4 min after the sample solution reached 37 and 43 °C, respectively. In the first morphological transition from MVVs to OLVs, a straightforward speculation about the mechanism of the transition would be the fusion inside each individual MVV, resulting in the inner bilayer of an OLV. The sample adopted a surprisingly unexpected process. Immediately after the temperature arrived at 37 °C at a heating speed of 6 °C/min, we already found the presence of the OLVs and also a significant number of intermediate vesicles of larger dimensions (larger than 500 nm) with many complex structures inside (Figure 5b, 0 min). This showed that fusion between different MVVs took place, resulting in intermediates with larger sizes and internal vesicles. After 1 min at 37 °C, a clear transition followed by elongation was found with a lot of tubular vesicles of lengths larger than 500 nm. It is noteworthy that all tubular vesicles showed multiple stacked bilayers (mostly double bilayers) (arrows in Figure 5b, 1 min). The outmost bilayer in vesicles served as a templating membrane to guide the fusion process. After 4 min at 37 °C, we found only OLVs, which indicates the transition completed. A fission process was responsible for the transition between the elongated tubular vesicles and OLVs. In the second morphological transition from the OLVs to the MLVs, increasing the temperature to 43 °C for 1 min already triggered the transition (Figure 5c, 1 min). We observed a mixture of initial OLVs (white arrow in Figure 5c, 1 min) and an immature form of MLVs (blue arrow in Figure 5c, 1 min). A new type of OLV (yellow arrow in Figure 5c, 1 min) was also observed with discernible bilayers, which is different from the stacked bilayers in initial OLVs. These OLVs are an intermediate form between initial OLVs (white arrow) and immature MLVs (blue arrow). The presence of the new type of OLVs and immature MLVs indicates both fission and fusion occurred during the transition. Extending the incubation time to 4 min at 43 °C completed the transformation to MLVs (Figure 5c, 4 min). These rapid morphological transitions were believed to be attributable to their high molecular flexibility and dynamicity, similar to lipids, stemming from their relatively low molecular weight and molecular design.

### 2.4 Energy Landscapes Navigated by Molecular Design

We have demonstrated great variability in vesicular states at different temperatures (Figure 4b). Of particular importance is the ability to control the pathway between uniform OLVs and MVVs at R.T. (Figure 4c). It would be even more exciting if such pathway selectivity happens at a body temperature of 37 °C, where dendrimersomes find their great potential for biomedical applications such as antibacterial nanoreactor^26^ or targeted mRNA delivery.^27–29^ To investigate this concept, we set out to encode the dependence of vesicular structure on the pathway in the design of Janus molecules.

The hydrophilicity of OEG or its counterpart with more units of ethylene glycol of poly(ethylene glycol) (PEG) has been well acknowledged and used as the hydrophilic block to construct self-assemblies.^30^ The oxygen atoms of OEG chains could preferably interact with protic solvent molecules (e.g., water) by forming hydrogen bonds, which contributes to their well-hydrated feature.^31,32^ However, the intermolecular dipole–dipole interaction could also drive the interdigitation of OEG chains in toluene.^18^ The interdigitation of the OEG in an aqueous medium has not been fully explored, particularly in hierarchical self-assembly systems. In a previous extensive study,^21^ it has been proposed that the branching pattern of Janus dendrimers would have an effect in the size and mechanical property of dendrimersomes, which resulted from the interdigitation of hydrophobic alkyl chains. From the XRD and simulation studies, the (3,5)-positioned pattern of the hydrophobic part allowed more interdigitated packing of the aliphatic chains than its (3,4)- and (3,4,5)-positioned counterparts. We postulate that the current (3,5)-branching pattern of OEG provides a structural premise for the interdigitation as shown in bulk.^21^ Upon self-assembly as prepared by the fast injection, kinetically trapped nonconcentric MVVs were obtained with only partial interdigitation of the OEG of adjacent bilayers present, as indicated in the cryo-TEM images. A non-negligible feature of the nonconcentric MVVs after self-assembly is that nearly all MVVs showed “intra-attraction” in some portion of the bilayers, either in the presence (Figure 2a) or absence of ethanol (Figure 3e). Specifically, internal vesicles in an individual MVV exhibited attachment to the relatively outside vesicle, as represented by a portion of the vesicle with a thicker membrane in appearance. The attachment between bilayers indicates the interdigitation of the hydrophilic corona, i.e., the OEG corona. This attachment among vesicles resulted in the unique nonconcentric feature of the MVVs (Figure S13a). When internal vesicles attached to their outer bilayer vesicles completely, the appearance of a thicker membrane was observed (Figure S13b).

We next probed the chain mobility of the self-assemblies by ^1^H NMR to help explain the morphological transitions at different temperatures (Figure S14). The OEG units in the vesicles were quite rigid due to their short chain length, as demonstrated by the invisibility of the OEG units in ^1^H NMR at R.T. Upon increasing the temperature from R.T. to 37 °C, the favorable interaction between the –OCH_3_ end groups or between the –OCH_3_ end groups and –OCH_2_CH_2_– groups may lower the free energy of the system. This resulted in the complete interdigitation of OEG chains between bilayers, as presented in OLVs. At 37 °C, OEG units displayed limited mobility with broad peaks^33^ in ^1^H NMR due to the interdigitation. Further increasing temperatures to 43 and 50 °C, the NMR spectra showed sharp OEG peaks due to their high mobility, suggesting the disappearance of the OEG interdigitation. The molecules became greatly flexible where the aliphatic and even aromatic moieties were visible. Together with the structures as observed from cryo-TEM, the OLVs transform to MLVs with the disappearance of the interdigitation of OEG chains, as shown by the detachment between bilayers of the MLV (43, 50, and 60 °C in Figure 4b). We propose two reasons for such a phenomenon. First, the increased temperature tilted the balance to entropy, which outperformed the favorable interaction between the end groups of the OEG at higher temperatures. Second, the middle linking part of the dendrimer molecule is constituted by carbon–carbon single bonds, which possess high rotational freedom (Figure 1a). At higher temperatures, the rotation of these single bonds could take place, resulting in the flip of the dendrimer molecules,^22^ which could disrupt the interdigitation of the OEG chains. The disappearance of interdigitation of bilayers in the OLVs resulted in the transformation to MLVs. Notably, comparing with the OLVs annealed from 37 °C (R.T. II in Figure 4c), this new state of MLVs could guide the system to a different pathway when cooling back to R.T., leading to the formation of kinetically trapped MVVs again (R.T. III in Figure 4c).

**Figure 6:**
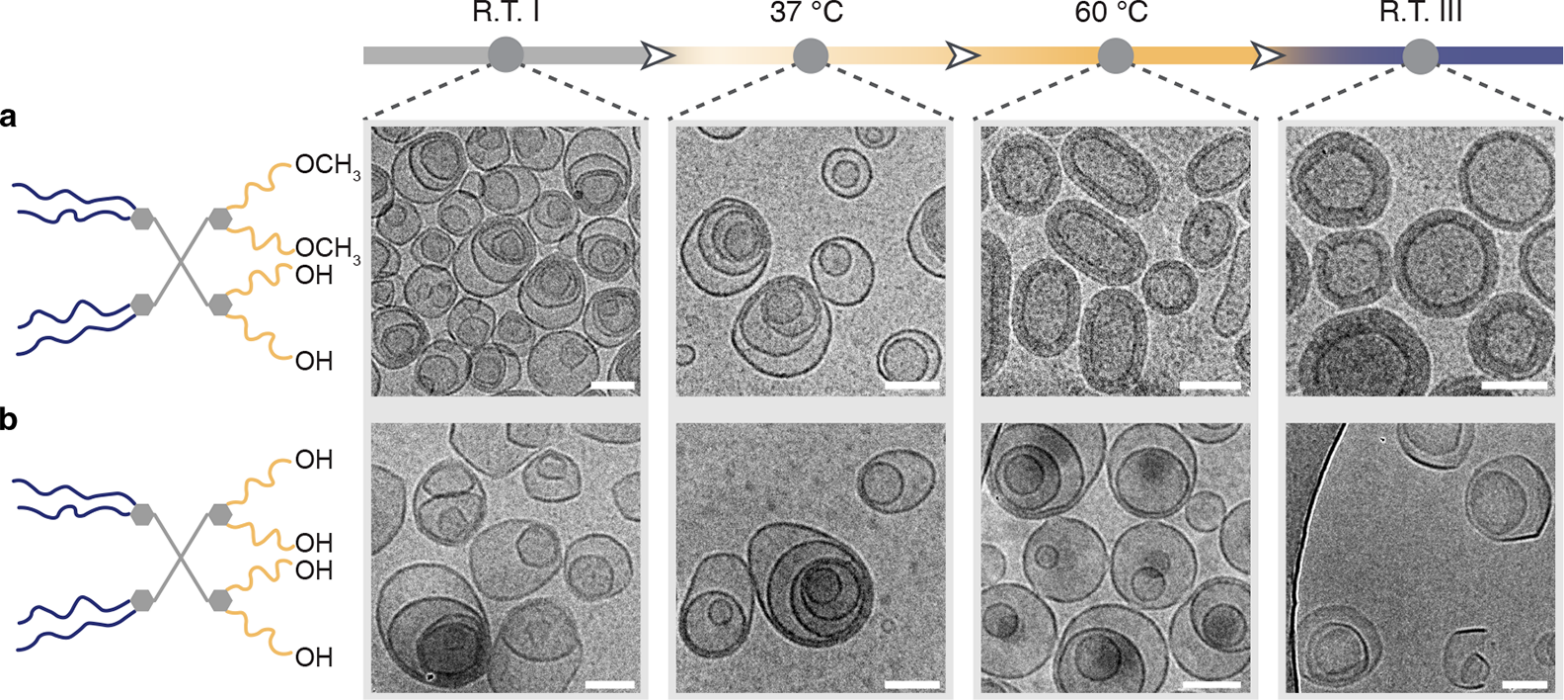
Assessment of the navigation of energy landscapes of assemblies with different end groups. Cryo-TEM images of self-assemblies as vitrified at indicated temperatures during the first heating/cooling cycle. (a) Vesicles self-assembled from Janus dendrimers with half the methoxy and half the hydroxy end groups per molecule at different temperatures. (b) Vesicles self-assembled from Janus dendrimers with hydroxy end groups at different temperatures. Scale bars are 100 nm.

The methoxy (–OCH_3_) end group of the OEG in our Janus dendrimer displays relative hydrophobicity in the OEG. We hypothesized that changing the –OCH_3_ end groups can shift the critical temperature, which triggers the interdigitation of the OEG. This is envisioned to achieve a pathway selection between vesicles at temperatures other than R.T. in the previous system. To further substantiate this hypothesis, we synthesized the other three types of Janus dendrimers with exactly the same molecular structure except for the end groups of OEG chains. The original dendrimer molecule had four –OCH_3_ end groups per molecule (Figure 1c). To alter the hydrophobicity of the end groups of the OEG, we gradually replaced the four –OCH_3_ groups per molecule with two and four –OH groups, rendering the molecules with increased hydrophilicity of the end groups (Schemes S2–S4). The molecular dynamics simulation further confirmed the higher degree of hydrophilicity exhibited by the OEG of the –OH end-group dendrimer when compared to that of the –OMe end-group dendrimer across all temperatures investigated in the study (Figure S15 and Table S1). The monodispersity of the molecules was confirmed by NMR and MALDI-TOF (Figures S16–S18), followed by the same self-assembly procedure. It is worth mentioning that the unique monodisperse molecules make it possible to investigate only the effect of the end groups in energy landscapes while keeping other parameters exactly the same. As demonstrated by DLS, self-assemblies composed of the newly synthesized dendrimers after dialysis displayed similar size and PDI as compared with the previous sample made from dendrimers of four –OCH_3_ end groups (Figure S19). Moreover, all assemblies also exhibited MVV morphologies as shown by cryo-TEM (Figures 6 and S20, R.T. I). This indicated that altering end groups from –OCH_3_ to –OH exerted a limited influence on the self-assembly of the investigated dendrimers with the same branching patterns. Next, we examined the energy landscapes of these three systems by bringing them to different temperatures. In particular, we chose two equilibrating temperature points of 37 and 60 °C to compare with the previous results. As expected, there was no transition of MVVs at 37 °C of the sample replaced with half of the –OCH_3_ end groups (i.e., two –OCH_3_ and two –OH end groups per molecule, Figure 6a). This was due to the increased hydrophilicity conveyed by the –OH end groups. As a consequence, equilibration at 37 °C could not provide enough energy to induce the interdigitation of the OEG chains as compared with that of all –OCH_3_ groups, as shown in the previous section. This energy barrier could be surpassed by bringing the system to a higher temperature. After equilibrating at 60 °C, a similar transition between MVVs to OLVs was observed, indicating the occurrence of OEG interdigitation. This state of the OLVs remained unchanged after cooling to lower temperatures such as 37 °C or R.T., which is reminiscent of the first pathway of –OCH_3_ end-group assemblies annealed from 37 °C (Figure 4c). We also showed that the position of the –OCH_3_ and –OH end groups, either from the same dendron (Figure S17) or vice versa (Figure S16), exerts minimal influence on their self-assembly behaviors as well as the morphological transition at 60 °C (Figure S20). We further checked the same temperature treatment for assemblies with complete –OH end groups of the dendrimer. Dendrimers with –OH end groups have the highest hydrophilicity among all three types of dendrimers. We envision that the interdigitation of OEG chains will become challenging. Consistent with the expectation, the original MVVs remained throughout all temperatures from R.T. to 60 °C (Figure 6b), confirming that the studied temperature range could not induce any further indigitation of the OEG chains.

By molecule design, we show that we can navigate assemblies into different energy wells (Figure S21). The trigger of the interdigitation of the OEG could be tuned and shifted to higher temperatures by encoding the amphiphilicity of –OCH_3_ to –OH end groups. Noteworthily, the assemblies of half –OH end groups per molecule display a switch between distinct vesicles at the biomedically relevant temperature of 37 °C. The assemblies could be programed to be either MVVs (bringing to 37 °C from R.T.) or OLVs (annealing to 37 °C from a higher temperature at 60 °C). The different morphologies and conformations of the OEG corona (interdigitated for OLVs versus noninterdigitated for MVVs) at 37 °C might offer distinct properties of the assemblies in the biomedical realm. The other interesting finding is that although the assemblies of half –OH end groups delayed the interdigitation process of the OEG, once formed, the interdigitation is more stable than that of all –OCH_3_ end-group assemblies. The interdigitation of the OLVs of –OCH_3_ end-group assemblies was disrupted at 60 °C, forming MLVs (Figure 4b), while for the OLVs of half –OH end-group assemblies remained intact at 60 °C (Figures 6a and S20). We speculated that the hydrogen bonding of –OH groups in the interdigitation of OEG was responsible for their increased stability.

## 3 Conclusions

Our study has demonstrated that vesicular self-assemblies display a rich energy landscape with different thermodynamic wells of diverse vesicular morphologies depending on the environmental temperature and their thermal history. Additionally, the presence of solvent adds another possibility to render extra energy minima of the assemblies. Our results highlight the importance of the thermal history during the formulation of samples and the thorough characterization of the samples before their use for applications. The transitions of samples among different energy minima could be neglected in batch measurements such as DLS. The concept of energy landscapes and pathway complexity resonates with other supramolecular systems of fibers and is not commonly anticipated for artificial vesicular systems, despite their wide prevalence in cellular vesicles. In this context, we further revealed a strategy to navigate the pathway selection between distinct vesicular structures by molecular design to shift the balance of the competing interactions from the molecules. Notably, the interplay interaction derived from the hydrophilic corona of OEG of the vesicles highlights the complexity and function of OEG (or its counterpart of PEG), whose inert role has been challenged recently as a possible suspect for anaphylactic reactions to COVID-19 vaccines.^34^ To further substantiate the link of structural landscapes and functions, particularly in biomedical applications, as well as the potential role of OEGs with different states, molecules with half methoxy and half hydroxy end groups serve as an ideal model since their assemblies could display two different vesicular structures at 37 °C after performing the pathway selection via thermal treatment. We envision that the present strategy of tuning the competing interaction within molecules for a rich landscape of vesicular assemblies could be expanded to other types of Janus dendrimers, including Janus glycodendrimers,^35^ ionizable amphiphilic Janus dendrimers,^27–29^ and stereochemical Janus dendrimers.^36^

## 4 Experimental Section

### 4.1 Materials

All reagents are used as received without purification, unless otherwise indicated. 1-Bromododecane, pentaerythritol, and palladium on activated carbon (10% Pd, unreduced) were purchased from Acros Organics. Triethylene glycol monomethyl ether, triethylene glycol, *p*-toluenesulfonyl chloride, methyl 3,5-dihydroxybenzoate, benzaldehyde, 4-(dimethylamino)pyridine (DMAP), *p*-toluenesulfonic acid monohydrate, *N*,*N*′-dicyclohexylcarbodiimide (DCC), and benzyl bromide were products from Sigma-Aldrich. Dimethylformamide (DMF), potassium carbonate (K_2_CO_3_), sodium hydroxide (NaOH), sodium carbonate (Na_2_CO_3_), dichloromethane (DCM), and methanol were purchased from Thermo Fisher Scientific. Methyl 3,4-dihydroxybenzoate was bought from TCI Europe NV. Ethanol (EtOH) and tetrahydrofuran (THF) were products from VWR International. Potassium hydroxide (KOH) was bought from J.T.Baker, Avantor. Hydrochloric acid (HCl, 37%) and potassium iodide (KI) were purchased from Merck. Dry THF and dry DCM were obtained by passing solvents over activated alumina columns in a MBraun MB SPS800 under nitrogen and stored under argon. Ultrapure Milli-Q water (QPOD Milli-Q purification system, 18.2 MΩ) was used for the preparation of all nondeuterated aqueous solutions.

### 4.2 Janus Dendrimer Synthesis and Characterization

All Janus dendrimers were synthesized according to previously reported protocols.^19^ Their purities were determined by NMR as well as MALDI-TOF. Detailed description is available in the Supporting Information.

### 4.3 General Self-Assembly Preparation

Self-assembly was carried out by fast injection (∼0.5 s) of 100 μL of dendrimer solution in ethanol into 2 mL of Milli-Q water followed by 5 s of vortex mixing, resulting in a final concentration of ∼0.5 mg/mL. For samples of higher final concentrations, the injection process with the same volume of solution was performed by injecting the ethanol solution of dendrimer with higher concentrations correspondingly.

To remove the ethanol in the as-prepared sample, the sample solution was transferred into dialysis tubing of a spectra/Por dialysis membrane (molecular weight cutoff: 3.5 kDa) and dialyzed against a large amount of Milli-Q water with frequent changes of medium under continuous stirring.

### 4.4 Cryogenic Transmission Electron Microscopy

#### 4.4.1 Cryo-TEM Imaging at Room Temperature

Cryo-TEM imaging was carried out with a JEOL Transmission Electron Microscope 2100 at 200 kV with a high-quality Gatan 895 ultrascan 4000 bottom mount camera (4080 × 4080 pixels) incorporated to capture the morphologies of self-assemblies. TEM grids (Quantifoil) were glow-discharged by a 208-carbon coater (Cressington). To image a sufficient amount of particles, the original sample solution was concentrated by centrifugation. Then, 3.5 μL of sample solution was loaded onto the grid, blotted, and then vitrified through plunging into liquid ethane at 100% humidity with FEI Vitrobot Mark IV (blot time 1.5 s, blot force 2). Samples were loaded in a 914 high-tilt cryoholder (Gatan, Munich, Germany) and inserted into the microscope for imaging. Data analysis was performed using an open-source image processing software, Fiji ImageJ (v 2.1.0).

#### 4.4.2 Cryo-TEM Imaging at Higher Temperatures

To capture the morphology of the sample in the heating/cooling cycles, cryo-TEM imaging above room temperature at 37, 43, 50, and 60 °C was performed under modified procedures, according to a recently published report.^25^ Briefly, the Vitrobot chamber was set to the studied temperature and 100% relative humidity. At high temperatures, high water vapor content could cause water condensation, resulting in many challenges in the operation for sample preparation as well as in obtaining grids with optimum ice thickness for imaging. To minimize the water condensation at high temperatures, a homemade device from a falcon tube was installed to direct the flow of water vapor. Together with the tiny clamping contact area (as small as possible to freeze the sample effectively), the success rate to obtain sample areas with thin ice thickness when imaging was significantly improved. The heating/cooling cycles were carried out with a programed temperature trend of controlled temperature ramping speed using the Apollo Thermal Cycler (model ATC401). The sample was equilibrated for 10 min at each temperature before being vitrified for imaging. The Eppendorf tips were placed on a hot plate to be preheated at indicated temperatures to avoid lowering the temperature of the sample when loading the sample onto grids. Tweezer/grid was uploaded into the Vitrobot chamber and incubated for 2 min at each temperature. Then the sample preparation was initiated following the standard procedure with modifications: loading 6 μL of sample solution onto the grid, waiting time for 2 s, followed by blotting, and then vitrification in liquid ethane (blot time 1.5 s, blot force 0). Samples were transferred to the holder and imaged with the same settings as described in the previous section.

#### 4.4.3 Quantification of Vesicles

Samples were checked with cryo-TEM and the number of different types of vesicles (see Figure S2) was counted for each sample. Images from different areas and different batches of samples were taken and counted to minimize the error. On average, images of each condition from different parts were randomly taken, and more than 500 particles were counted. The percentages for each type of vesicles were averaged, and the results were presented as bar charts, illustrating the frequency distribution of each morphology.

### 4.5 Cryo-TEM Image Processing

#### 4.5.1 Image Processing: 3D Surface Plots

To clearly distinguish morphologies of different kinds of vesicles, 3D surface plots were extracted from cryo-TEM images by using Fiji ImageJ as reported before.^37^ The Interactive 3D Surface Plot plugin converts pixel values to height information, resulting in 3D plots of the vesicles.

#### 4.5.2 Image Processing: Bilayer Thickness Quantification

Measuring membrane thickness by drawing a line plot manually may cause bias due to subjective selection. Moreover, the limited measurements could not give a whole picture of the distribution of the thickness of bilayers. Herein, we used Fiji ImageJ to map the thickness of the vesicular membrane in a large area. Therefore, we could obtain the distribution of the thickness of large quantities of vesicles (*n* > 50). Following the import of original cryo-TEM images into Fiji, the background of the image was inverted to a black background. “Gaussian Blur” and then “Subtract Background” were employed to reduce the background noise. Bilayer regions were then selected by adjusting “Threshold”. For different samples, similar threshold values were used. Regions of vesicular bilayers were chosen and added to “ROI manager” after setting the minimal value of 200 nm^2^ in “Analyze Particles”. This minimal value is to exclude any speckles in the background. The gray values of the selected bilayer region were further converted to length (i.e., membrane thickness) using the “Local Thickness” function, resulting in the distribution of the membrane thickness of the whole sample. A typical image after applying the processing procedures is shown in Figure S22, where the distribution of the membrane thickness of selected particles could be plotted as shown in the results of the article.

## Supporting Information Available

Additional experimental procedures, synthesis, and characterization of self-assemblies, including NMR spectroscopy, cryo-TEM, and DLS (PDF)
