# Supplementary material for: Complex Energy Landscapes of Self-Assembled Vesicles
Source: J Am Chem Soc. 2023 Jul 10;145(28):15496–506. doi: 10.1021/jacs.3c04285 (PMC10360149; doi:10.1021/jacs.3c04285)
Supplement: Supplementary file 1 — ja3c04285_si_001.pdf [file ja3c04285_si_001.pdf]

Supporting Information

Complex Energy Landscapes of Self-Assembled Vesicles

Jiabin Luan[1], Danni Wang[1], Shaohua Zhang[1], Yusuke Miyazaki[2], Wataru Shinoda[2], and Daniela A. Wilson[1]*

[1] Radboud University Nijmegen, Institute for Molecules and Materials, Heyendaalseweg 135, 6525 AJ, Nijmegen, The Netherlands

[2] Research Institute for Interdisciplinary Science, Okayama University, Okayama 700-8530, Japan

*Corresponding author. e-mail: d.wilson@science.ru.nl

Table of Contents

S1 | Experimental: synthesis 3

S1.1 | (3,4)12G1-PE-(3,5)-3EO-G1-$(OCH_3)_4$ 3

S1.2 | (3,4)12G1-PE-(3,5)-3EO-G1-$(OH)_4$ 5

S1.3 | (3,4)12G1-PE-(3,5)-3EO-G1-$(OCH_3)_2/(OH)_2$ 6

S1.4 | (3,4)12G1-PE-(3,5)-3EO-G1-$(OCH_3/OH)_2$ 7

S2 | Methods of molecular dynamics (MD) simulation 8

S3 | Experimental: characterization 9

S3.1 | NMR Spectroscopy 9

S3.2 | Mass Spectrometry 9

S3.3 | Dynamic Light Scattering (DLS) 9

S3.4 | Nanoparticles tracking analysis (NTA) 9

S4 | Supplementary Figures & Discussion 11

S5 | References 36

S1 | Experimental: synthesis

S1.1 | (3,4)12G1-PE-(3,5)-3EO-G1-(OCH_3)$_4$

Scheme S1. Synthesis of (3,4)12G1-PE-(3,5)-3EO-G1-(OCH_3)$_4$ Janus dendrimer

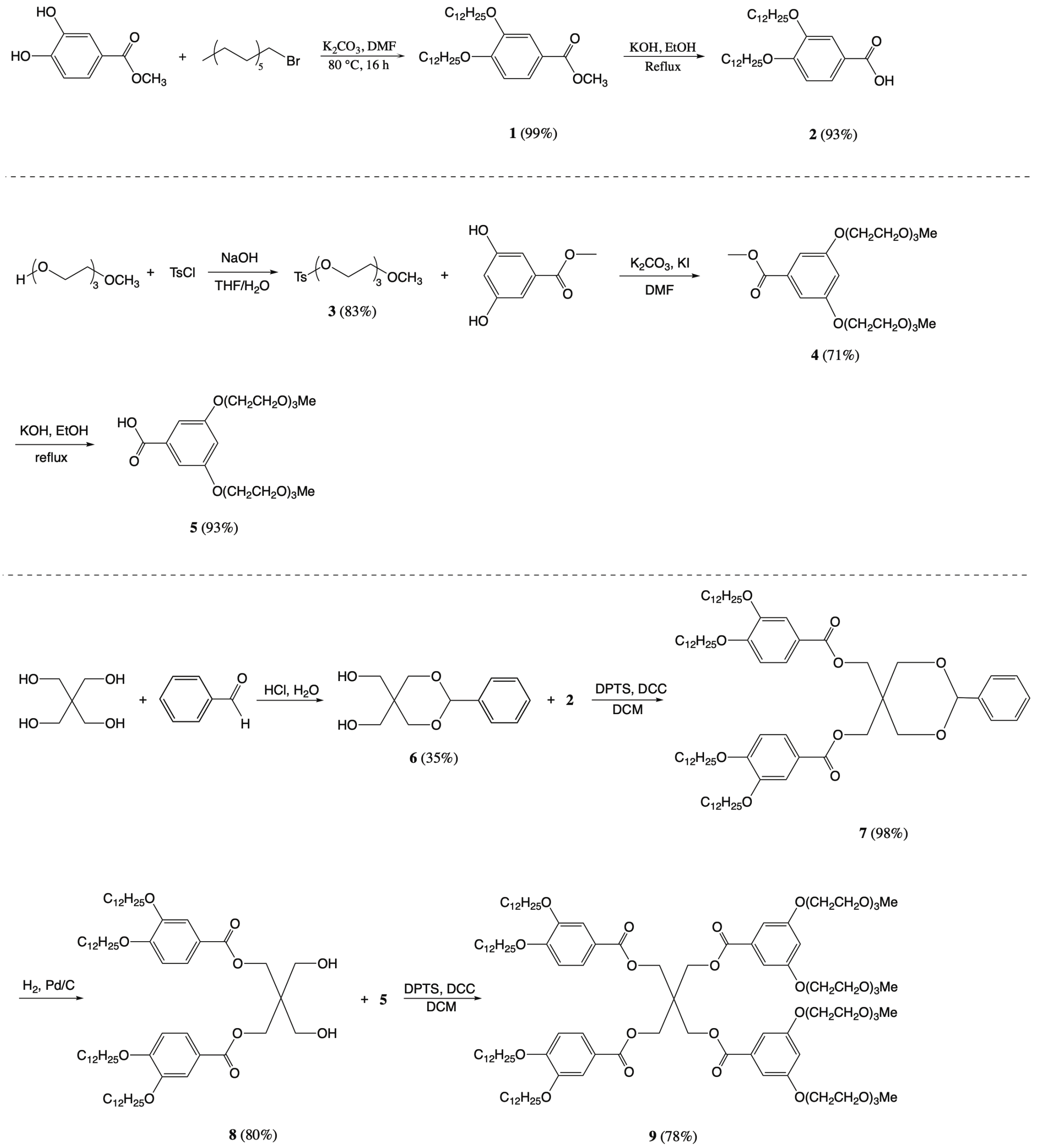

Amphiphilic Janus dendrimer ((3,4)12G1-PE-(3,5)-3EO-G1-(OCH_3)$_4$) composed of hydrophobic alkyl chains, hydrophilic oligo(ethylene glycol) and penthaerythritol core was synthesized in nine steps, as described previously.[1] In a nutshell, the hydrophobic unit was

synthesized convergently via direct esterification of 3,4-hydroxybenzoates with 1-bromododecane. The hydrophilic unit began with the tosylation of methyl-terminated triethylene glycol, followed by the reaction with methyl ester-protected gallic acid by Williamson ether synthesis. Corresponding acid was obtained by the removal of methyl ester group through hydrolysis. A benzylidene-protected strategy was used to differentially substitute the penthaerythritol core firstly with hydrophobic unit and then the hydrophilic unit. All products of each step were purified and characterized with nuclear magnetic resonance (NMR) and matrix-assisted laser desorption/ionization-time-of-flight (MALDI-TOF) mass spectrometry.

S1.2 | (3,4)12G1-PE-(3,5)-3EO-G1-(OH)4

Scheme S2. Synthesis of (3,4)12G1-PE-(3,5)-3EO-G1-(OH)4 Janus dendrimer

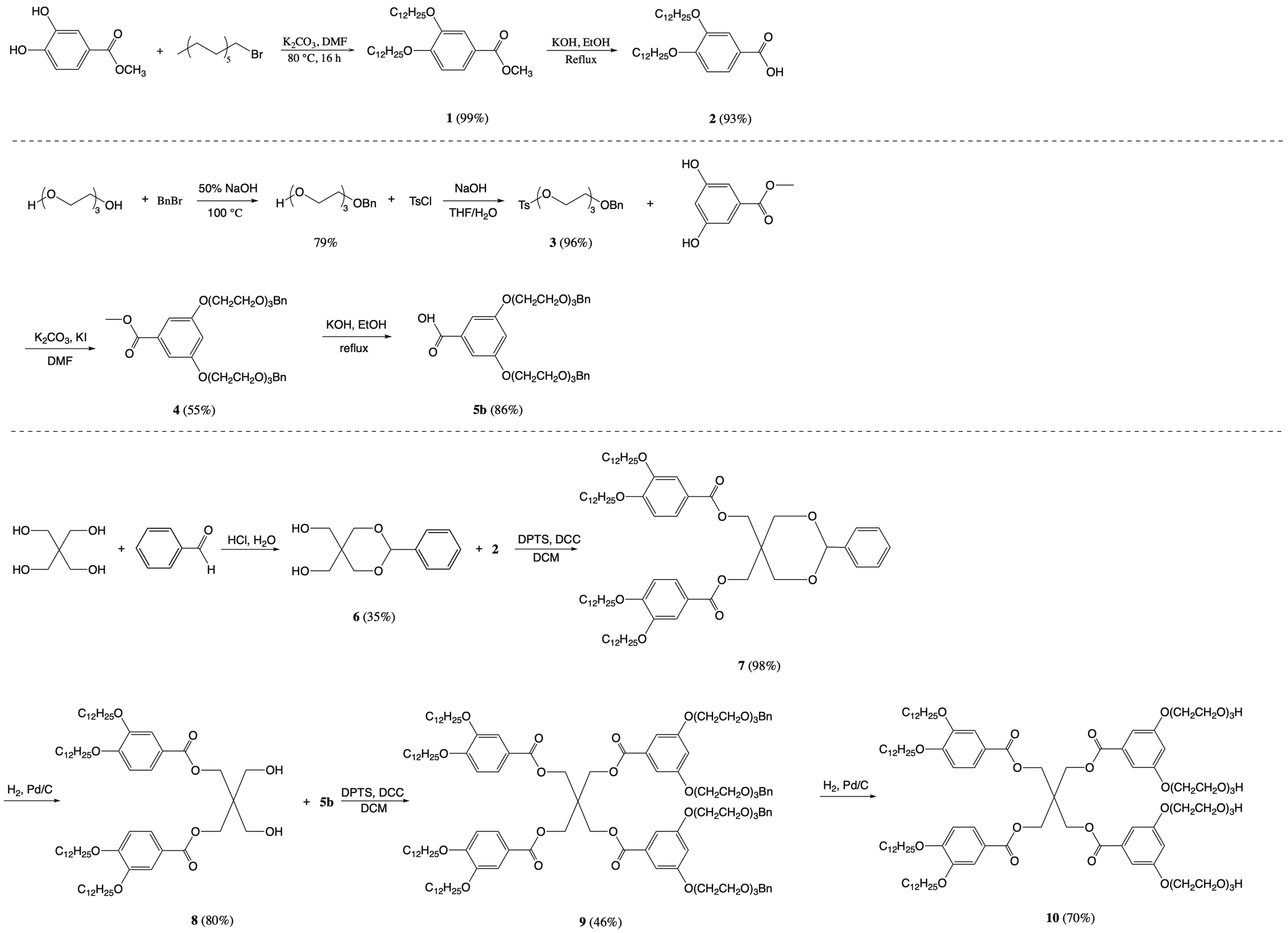

Synthesis of (3,4)12G1-PE-(3,5)-3EO-G1-(OH)4 proceeded similarly to (3,4)12G1-PE-(3,5)-3EO-G1-(OCH_3)4, except that tosylated triethylene glycol was protected with benzyl ether (Bn) (Compound **3**, **Scheme S2**). Williamson ether synthesis was carried out to incorporate the protected hydrophilic chains into the hydrophilic dendrons. Orthogonal synthesis by esterification was performed on the penthaerythritol core to obtain the Janus dendrimer. Finally, the protecting Bn groups were removed from the hydrophilic dendrons to give the hydroxy end-groups. All products of each step were purified and characterized with NMR and MALDI-TOF.

S1.3 | (3,4)12G1-PE-(3,5)-3EO-G1-$(OCH_3)_2$/$(OH)_2$

Scheme S3. Synthesis of (3,4)12G1-PE-(3,5)-3EO-G1-$(OCH_3)_2$/$(OH)_2$ Janus dendrimer

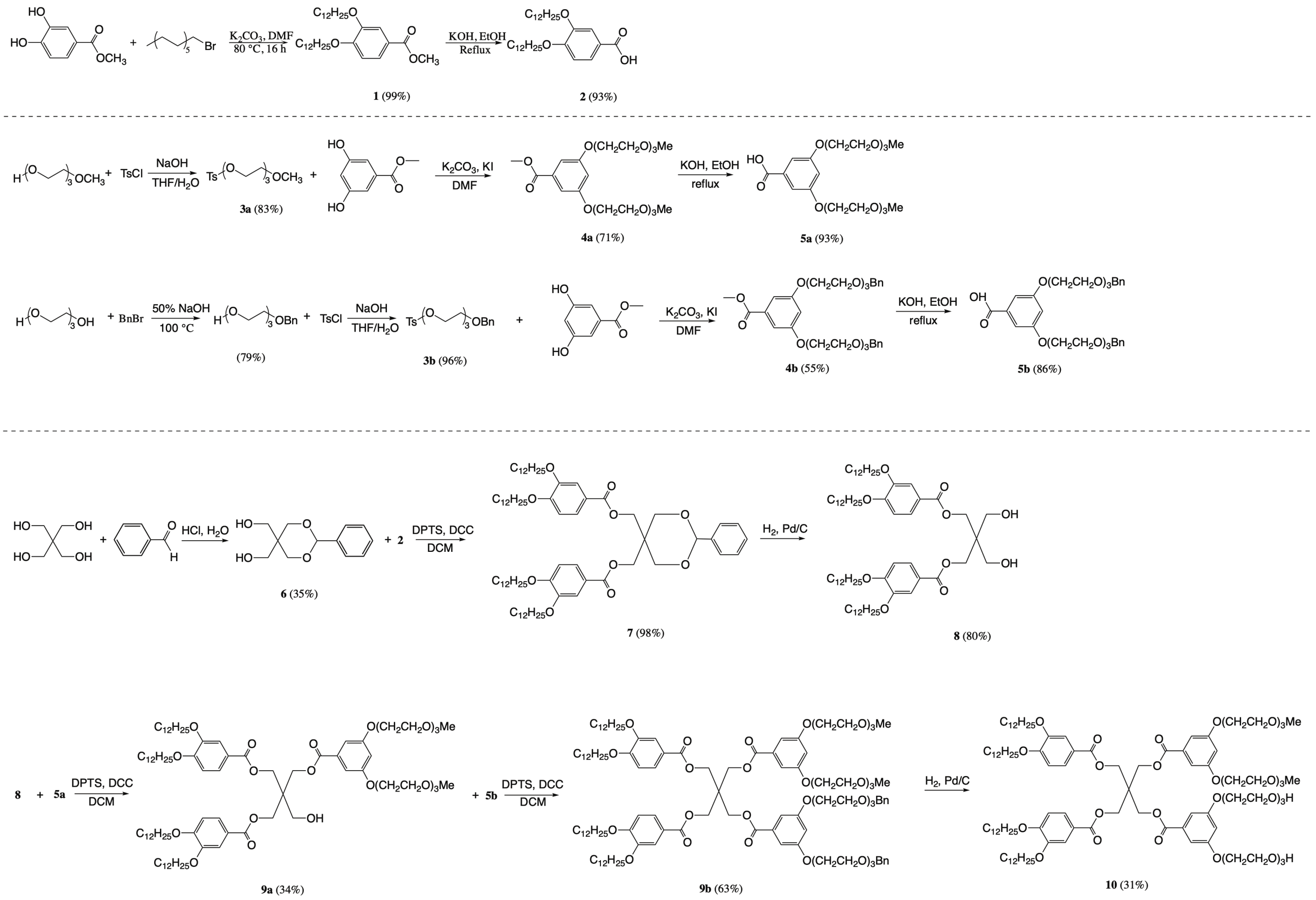

Synthesis of (3,4)12G1-PE-(3,5)-3EO-G1-$(OCH_3)_2$/$(OH)_2$ proceeded similarly to (3,4)12G1-PE-(3,5)-3EO-G1-$(OH)_4$, except that hydrophilic dendrons with -OCH_3 and -OH end groups were incorporated into the penthaerythritol core subsequently followed by the removal of Bn protecting groups (Compound **9a** and **9b**, **Scheme S3**). All products of each step were purified and characterized with NMR and MALDI-TOF.

S1.4 | (3,4)12G1-PE-(3,5)-3EO-G1-(OCH_3/OH)$_2$

Scheme S4. Synthesis of (3,4)12G1-PE-(3,5)-3EO-G1-(OCH_3/OH)$_2$ Janus dendrimer

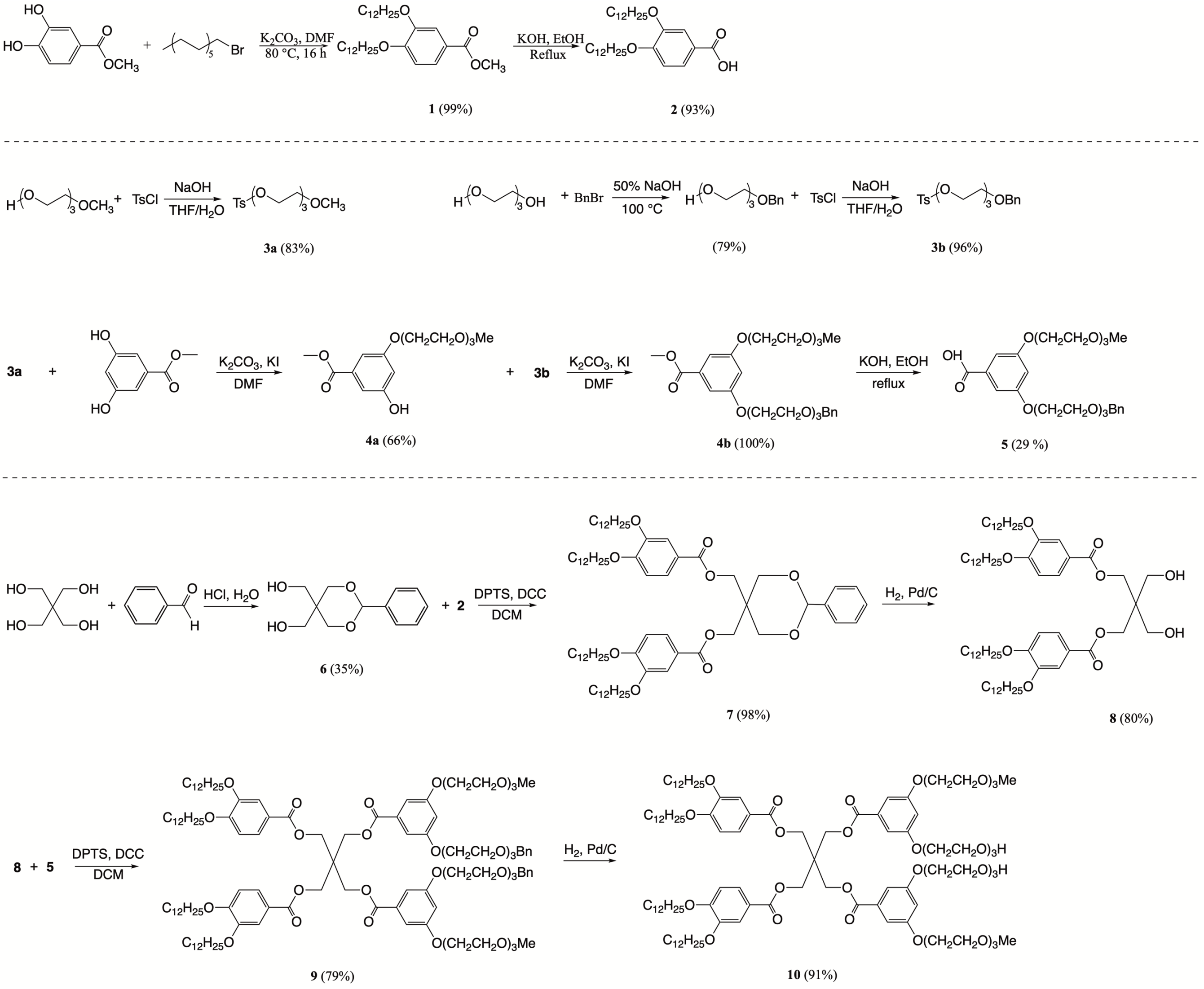

Synthesis of (3,4)12G1-PE-(3,5)-3EO-G1-(OCH_3/OH)$_2$ proceeded similarly to (3,4)12G1-PE-(3,5)-3EO-G1-(OCH_3)$_2$/(OH)$_2$, except that hydrophilic dendrons with -OCH_3 and -OH (first protected by benzyl group) end groups were incorporated into the same dendron (Compound **4a** and **4b**, **Scheme S4**). All products of each step were purified and characterized with NMR and MALDI-TOF.

S2 | Methods of molecular dynamics (MD) simulation

All MD simulations were conducted using the GROMACS 2021.5 package.[2] The CHARMM36 force field[3] with the TIP3P model[4] were adopted for the Janus dendrimers with complete −OMe or −OH end groups and water, respectively. The parameters of the dendrimers were assigned using CHARMM-GUI Ligand Reader & Modeler.[5] We prepared two simulation systems consisting of a fully hydrated single bilayer for each type of the dendrimer. The bilayer was composed 128 dendrimer molecules and was built using the MemGen web server.[6]

The system temperature mentioned below was maintained with a Nosé-Hoover thermostat.[7-8] A Parrinello Rahman barostat with semiisotropic coupling was used to control the system pressure to be 1 atm.[9] Electrostatic interaction was calculated using the particle mesh Ewald method.[10] The cutoff length of Lenard-Jones interactions was set to 1.2 nm with a force switching distance of 1.0 nm. The water geometries were maintained constant using the SETTLE algorithm.[11] All bonds involving hydrogen atoms were constrained employing the LINCS algorithm.[12] The simulation time step was set to 2 fs. Energy minimization with the steepest decent algorithm was conducted to prevent unstable structure in the initial configurations. We performed 200 ns MD simulations to equilibrate the single bilayer systems at 353 K. Then, further 100 ns equilibration runs were performed at three different temperature of 340, 310, and 280 K, respectively. We carried out 1 μs MD simulations of the systems as described above. The last 500 ns MD trajectories were used for analyses.

S3 | Experimental: characterization

S3.1 | NMR Spectroscopy

Nuclear magnetic resonance (NMR) spectra were recorded on a Bruker 400 MHz Avance III HD nanobay spectrometer with a BBFO probe. Tetramethylsilane ($\delta = 0.0$ ppm) or the residual protons of the deuterated solvent was used as the internal reference. ^{1}H NMR spectra were acquired using 32 or 64 scans and a relaxation delay of 5 s.

S3.2 | Mass Spectrometry

Matrix-assisted laser desorption/ionization time-of-flight (MALDI-TOF) mass spectrometry was performed on a Bruker Microflex LRF MALDI-TOF system equipped with a nitrogen laser (337 μm) and operating in reflection mode. Saturating matrix solution was prepared by dissolving α-Cyano-4-hydroxycinnamic acid in 30/70 (v/v) acetonitrile (ACN)/H_2O (0.1% v/v TFA) solution. The analytical sample was obtained by mixing a 1/1 (v/v) ACN/H_2O or THF solution of analyte (2 mg/mL). The solution of the analyte and matrix was mixed in equal ratio and 1.0 μL solution was loaded on the MALDI plate and allowed to dry at room temperature before inserting into the vacuum chamber of the MALDI instrument. The laser steps and voltages applied were adjusted accordingly depending on the nature of analyte.

S3.3 | Dynamic Light Scattering (DLS)

DLS measurements were carried out on Malvern Zetasizer Nano-ZS (Malvern Instruments) equipped with a He-Ne laser (633 nm, 4 mW) and Avalanche photodiode detector (173°) to evaluate the average hydrodynamic diameter (D_h) and polydispersity (PDI) of self-assemblies. For temperature trend measurements in heating/cooling cycles, samples were equilibrated for 300 seconds at each temperature before each measurement. Based on the Stokes-Einstein equation, the size and distribution of the particles were derived from the fluctuations in scattered light intensity due to the Brownian motion of the particles by assuming a hard sphere model.

S3.4 | Nanoparticles tracking analysis (NTA)

NTA was performed with Nanosight LM10-HS instrument with a Marlin camera and a 60 mW blue laser illumination (405 nm). NTA offers a particle-by-particle methodology with high

resolution results of the particle size and concentration. Typically, sample solution (0.5 mg/mL) was diluted by 1000 times to ensure an optimized number of particles (10^7 - 10^9 particles/mL) for analysis. The diluted solution was injected in a sample chamber. The Brownian motion of the nanoparticles was recorded in three videos of 60 seconds at 30 frames/s. The size and concentration of the sample were obtained by averaging the results as derived from the videos. Multiple measurements were conducted for each sample by the injection of sample solution.

S4 | Supplementary Figures & Discussion

a

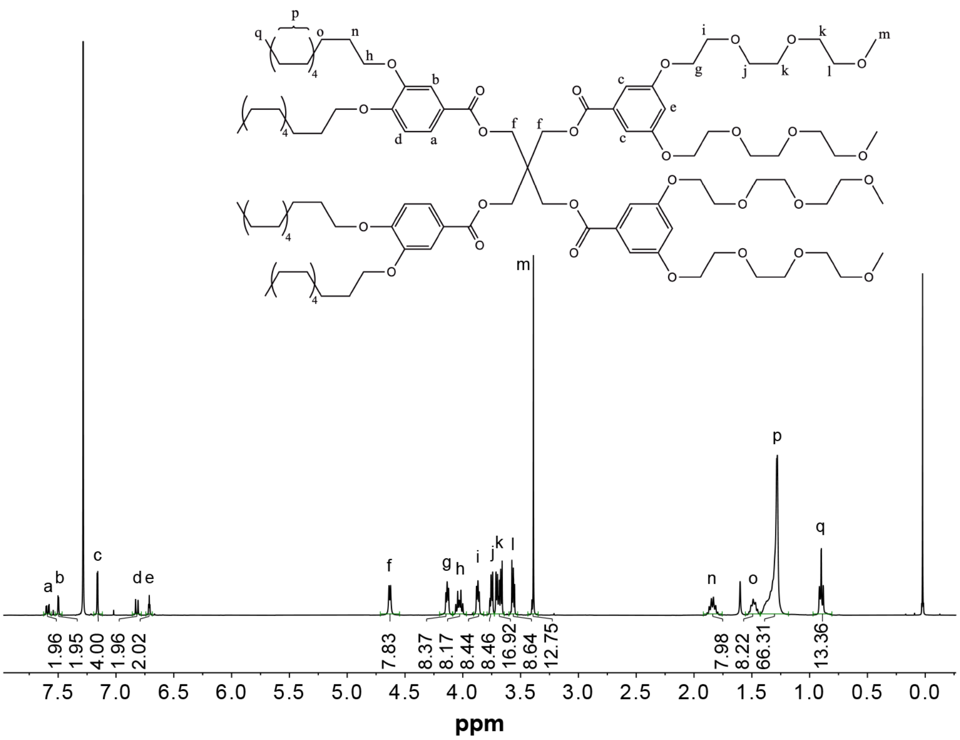

b

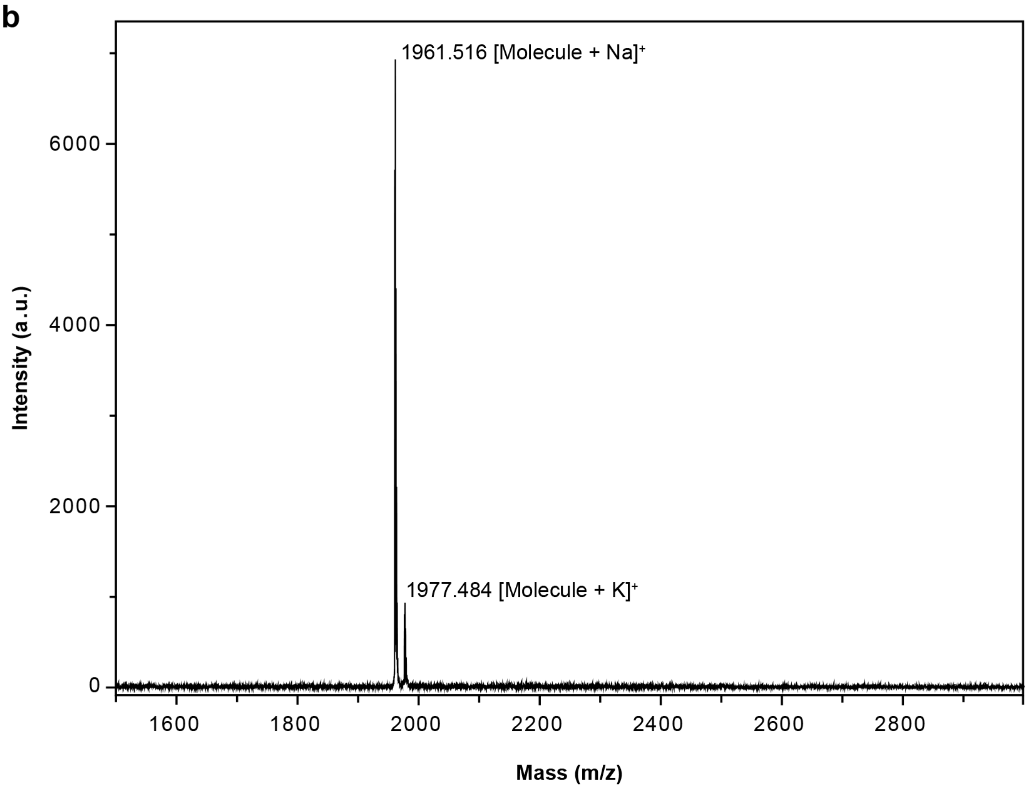

Figure S1. Characterization of (3,4)12G1-PE-(3,5)-3EO-G1-$(OCH_3)_4$ Janus dendrimer. (a) ^{1}H NMR in $CDCl_3$ and (b) MALDI-TOF spectra of (3,4)C12-PE-(3,5)-EG_3-$(OCH_3)_4$.

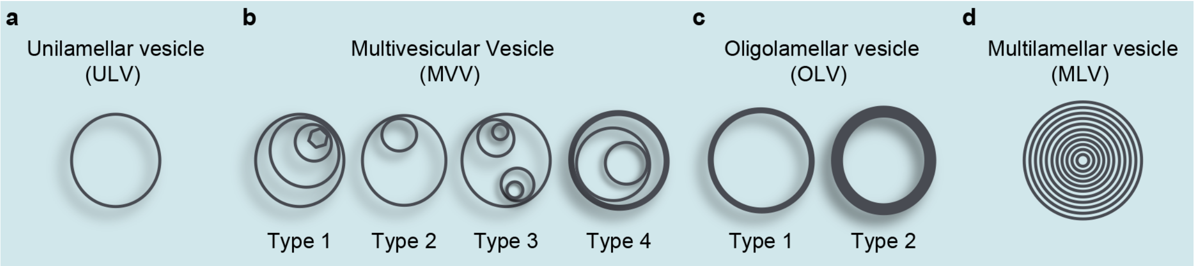

Figure S2. Typical vesicles involved in the current study. (a) Unilamellar vesicle (ULV) with single lamellae (bilayer). (b) Multivesicular vesicle (MVV) with nonconcentrically arranged internal vesicles. (c) Oligolamellar vesicle (OLV) with a few concentrically arranged internal vesicles. (d) Multilamellar vesicle (MLV) with many concentrically arranged internal vesicles, also known as onion vesicle.

Here vesicles are categorized following the same principle as reported in the well-established area of liposomes.[13] Among various types of vesicles, the most common and useful ones are vesicles with single bilayer (unilamellar vesicles, ULVs) or multiple bilayers. For vesicles with concentrically multiple bilayers, they are further classified into oligolamellar vesicles (OLVs) and multilamellar vesicles (MLVs, also known as onion vesicles) with only a few or many internal self-closed bilayers, respectively. Vesicles with nonconcentrically internal vesicles are called multivesicular vesicles (MVVs, also known as vesosomes), which could display in different types as shown in **Figure S2b**.

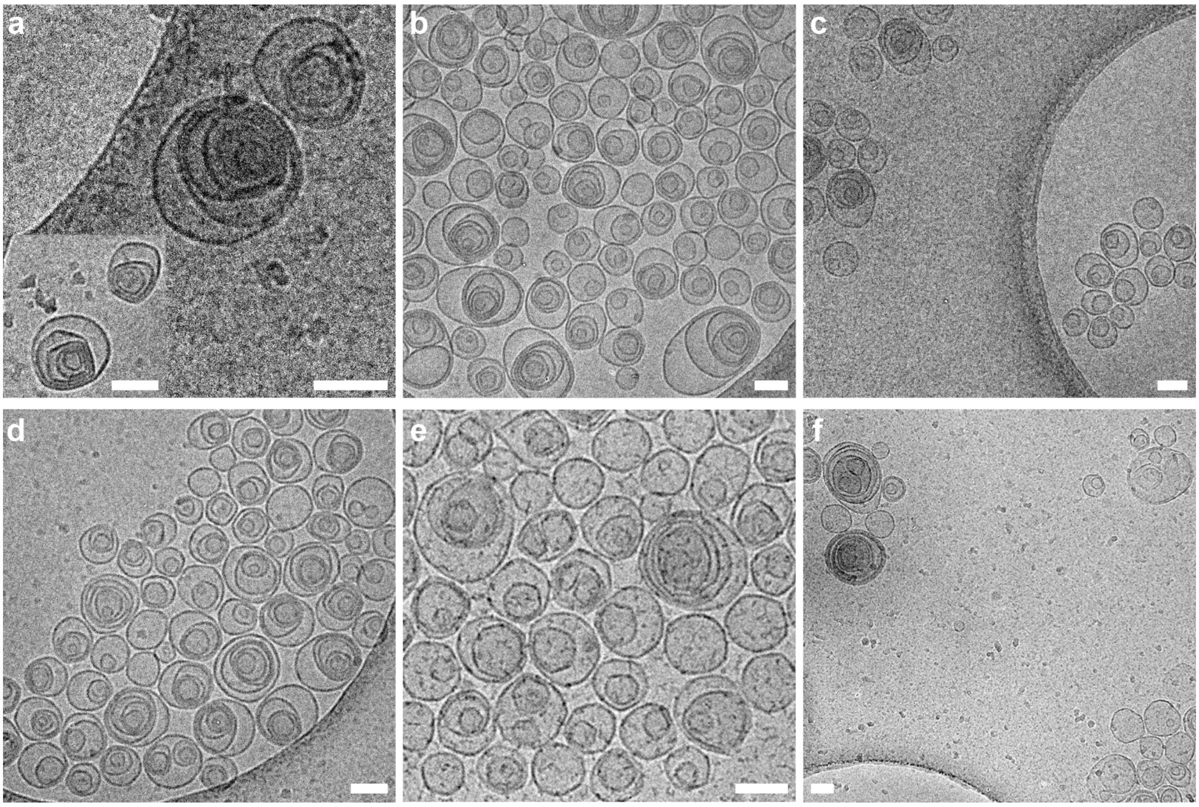

Figure S3. Effect of preparation conditions on the self-assemblies as characterized by cryo-TEM. (a) Self-assemblies prepared without centrifugation. (b) Self-assemblies prepared by injection of overnight-incubated dendrimer ethanol solution. (c) Self-assemblies prepared with low concentration of 0.2 mg/mL. (d) Self-assemblies prepared by injection of dendrimer isopropanol solution. e,f, Self-assemblies prepared by different injection speeds of dendrimer ethanol solution: ~0.2 s (e) and ~5 s (f). Scale bars are 100 nm.

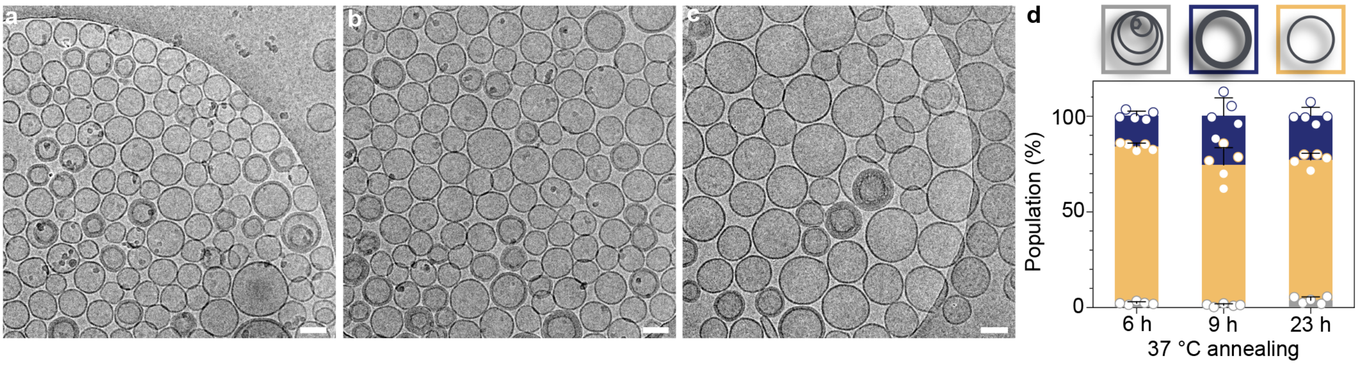

Figure S4. Stability of the structures of vesicles at extended annealing time from 37 °C. (a–c), Cryo-TEM images of self-assemblies annealed from 37 °C after being equilibrated for 6 (a), 9 (b), and 23 h (c). Scale bars are 100 nm. (d) Quantitative measurement of assemblies obtained from post-treatment at 37 °C. For each sample, images from different areas were taken and counted to minimize the error ($n > 500$ particles).

The size of the dendrimersomes was templated by the branching pattern of the Janus dendrimer molecules.[14] The size of the vesicles, as a result of the surface curvature, is therefore determined by the primary molecular structure in Janus dendrimers. In the current annealing experiments, the vesicles were self-assembled from the same molecule (i.e. (3,4)12G1-PE-(3,5)-3EO-G1-(OCH_3)$_4$). The branching pattern of the molecular structure remains the same under different temperatures. Therefore, the size of various vesicles did not change significantly. This explanation also accounts for the results in **Figure 3**e–g, where the size of vesicles in the absence of ethanol remained almost unchanged during their morphological transition.

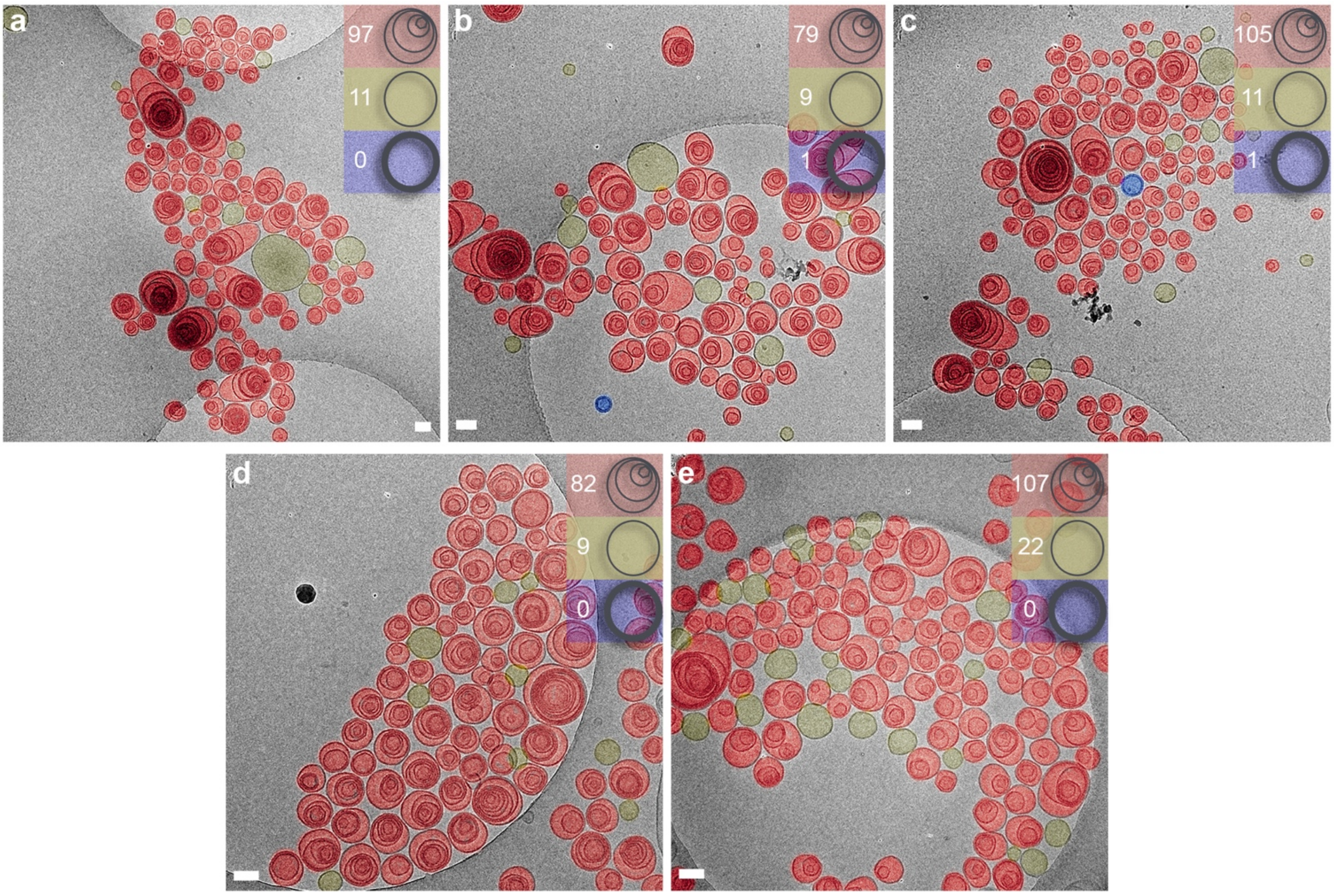

Figure S5. Quantitative measurement of assemblies obtained from direct injection in the presence of ethanol. Images from different areas and different batches of samples were taken and counted to minimize the error (n > 500 particles): (a–c) batch 1, (d) batch 2 and (c) batch 3. MVVs (red), ULVs (yellow) and OLVs (blue) are highlighted in false-colors. The insets of each image display the quantities of vesicles for each type. Scale bars are 100 nm.

We performed quantitative measurement of the percent frequency of each morphology for each condition (**Figure 2e**, **Figure 3h** and **Figure 4b**−the bar chart). Images from different areas and different batches of samples were taken and counted to minimize the error. In average, images of each condition were randomly taken and more than 500 particles were counted.

As an example, the analysis of images of self-assemblies prepared by direct injection in the presence of ethanol was shown in **Figure S5**. The percentages for each type of vesicles were averaged and the results were presented as bar charts, illustrating the frequency distribution of each morphology. The results derived from **Figure S5** correspond to the bar graph of 37 °C annealing for 0 h, as shown in **Figure 2e**. All other quantitative measurements were performed

in the same way.

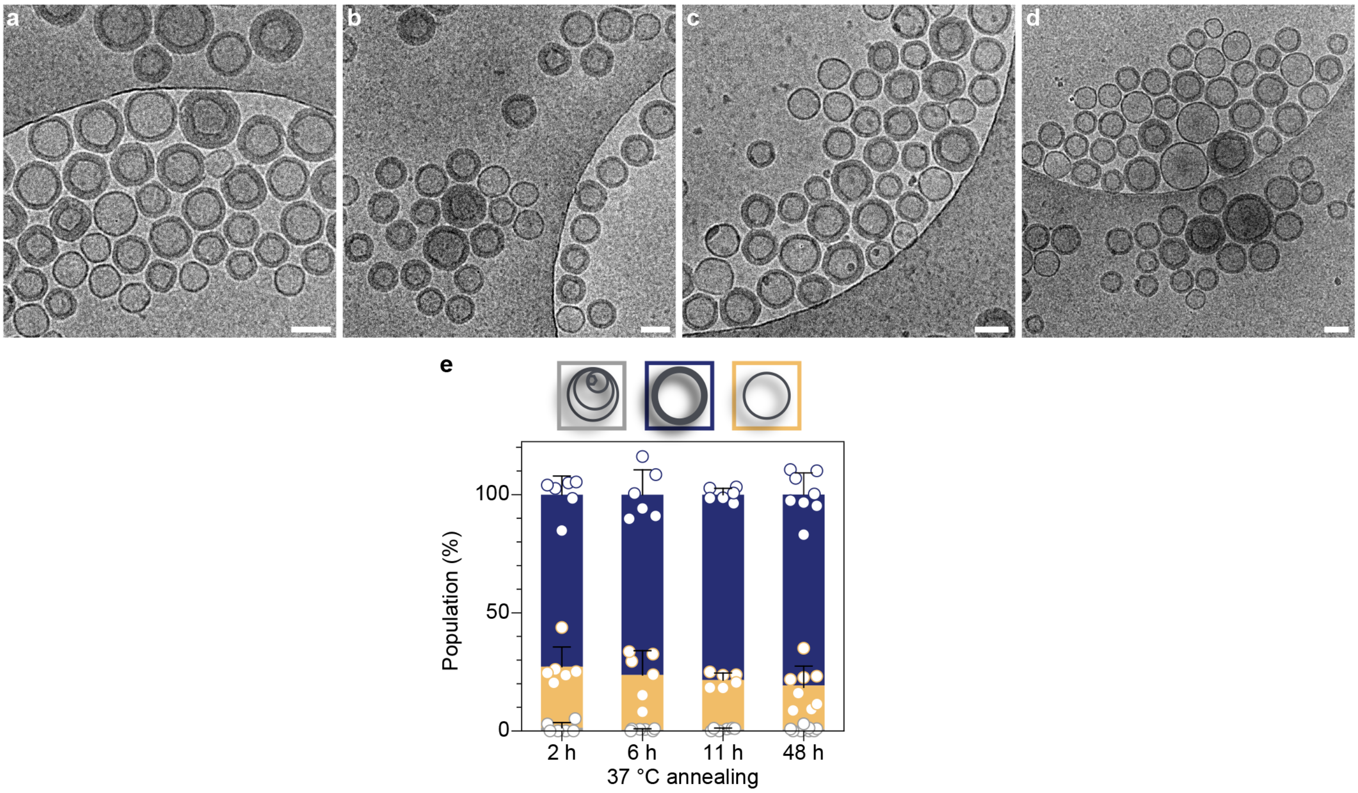

Figure S6. Stability of the structures of vesicles in the absence of EtOH at extended annealing time from 37 °C. (a–d) Cryo-TEM images of EtOH-removed self-assemblies annealed from 37 °C after being equilibrated for 2 (a), 6 (b), 11 (c), and 48 h (d). Scale bars are 100 nm. (e) Quantitative measurement of assemblies obtained from post-treatment at 37 °C. For each sample, images from different areas were taken and counted to minimize the error ($n > 500$ particles).

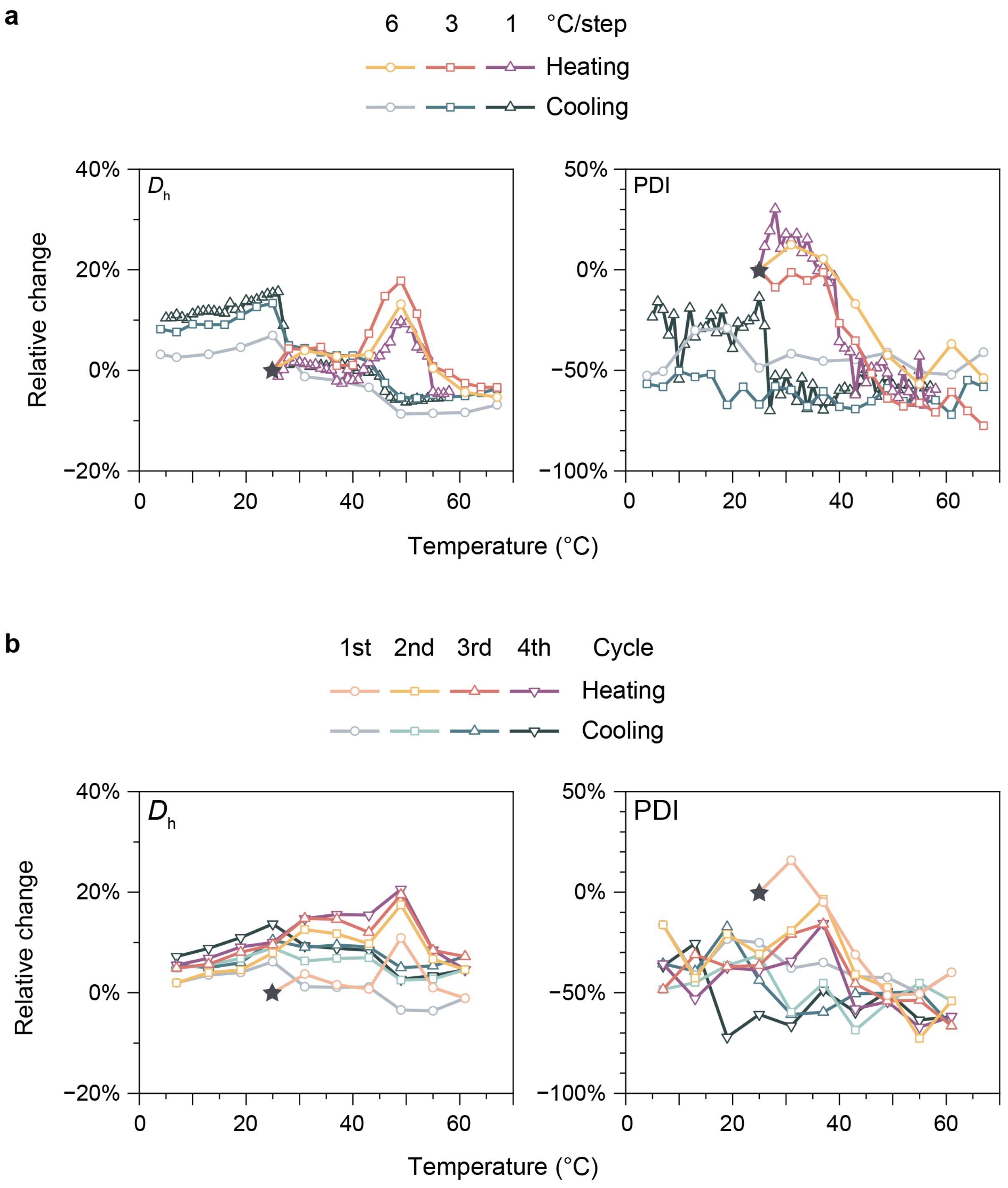

Figure S7. DLS characterization of self-assemblies in the absence of ethanol. (a) Relative change of D_h (%) and PDI (%) of assemblies at different heating/cooling rates in the DLS temperature trend measurements. (b) Relative change of D_h (%) and PDI (%) of assemblies at four heating/cooling cycles in the DLS temperature trend measurements.

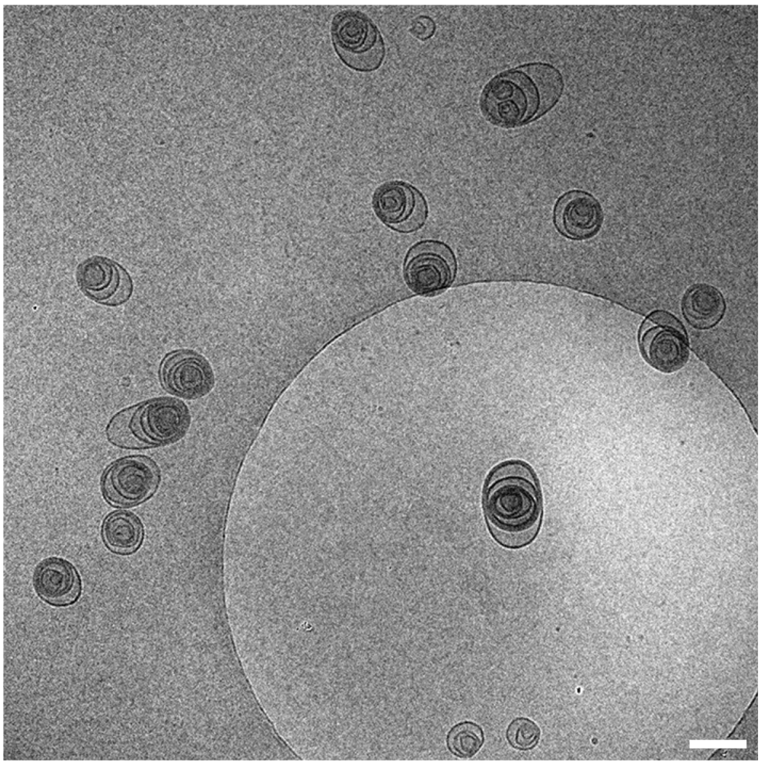

Figure S8. Cryo-TEM image of self-assemblies as prepared at 7 °C. Scale bar is 200 nm.

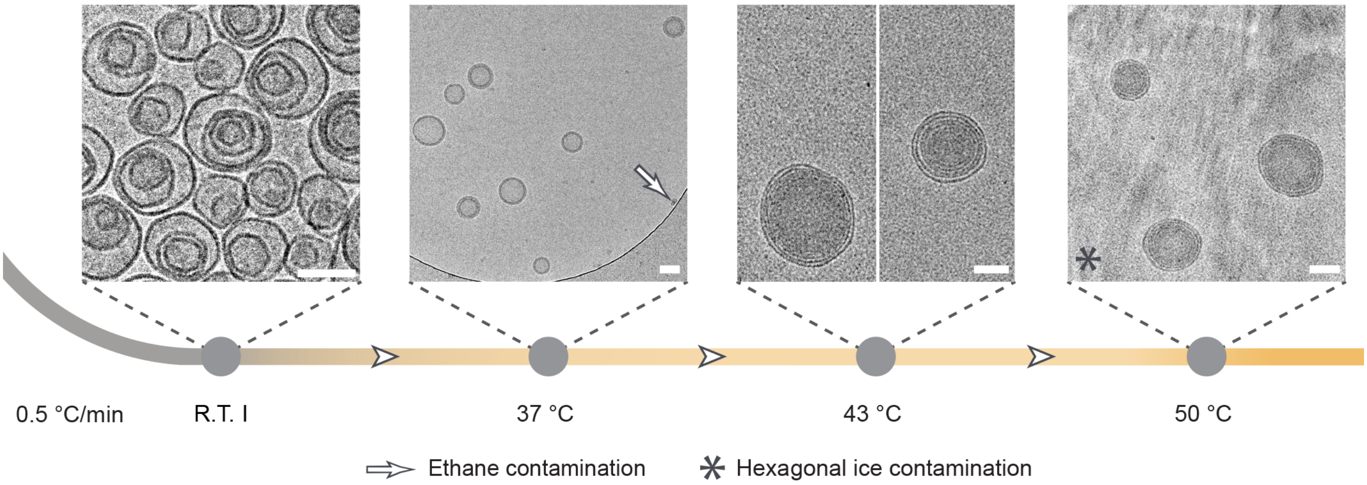

Figure S9. Effect of heating speed on the morphological transition of vesicles. Cryo-TEM images of self-assemblies as vitrified at indicated temperatures at the heating speed of 0.5 °C/min. Scale bars are 100 nm.

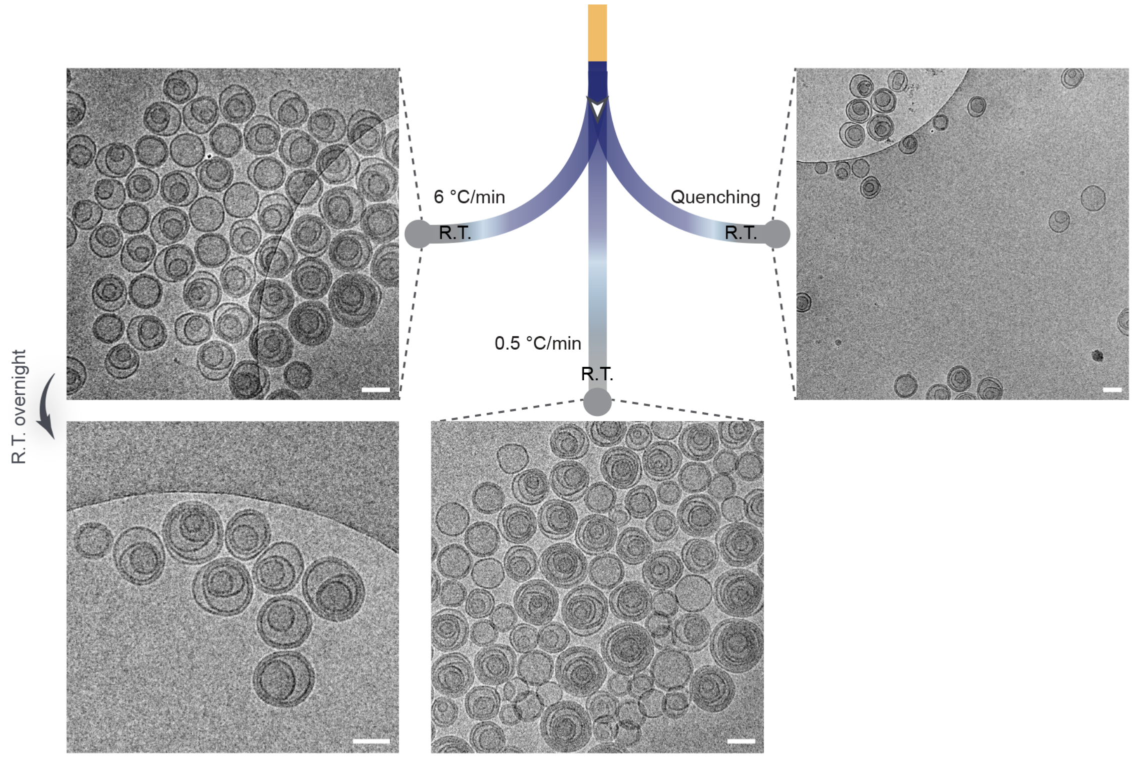

Figure S10. Effect of cooling speed from high temperature (43 °C or above) to room temperature on the vesicles. Scale bars are 100 nm.

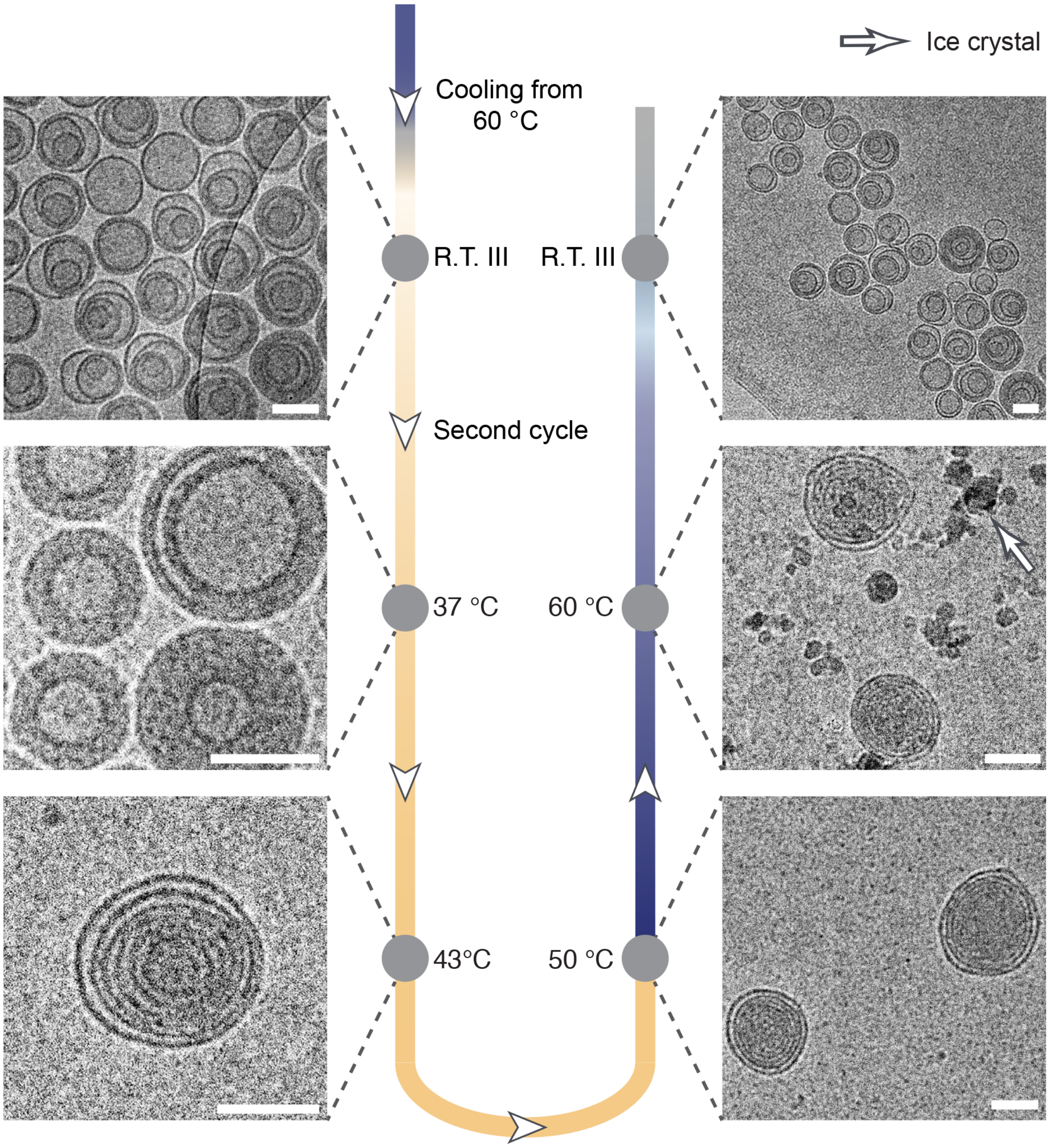

Figure S11. Morphological transition of vesicles (after the first heating/cooling treatment) in the second heating/cooling cycle. Cryo-TEM images of self-assemblies as vitrified at indicated temperatures during the second heating/cooling cycle. Scale bars are 100 nm.

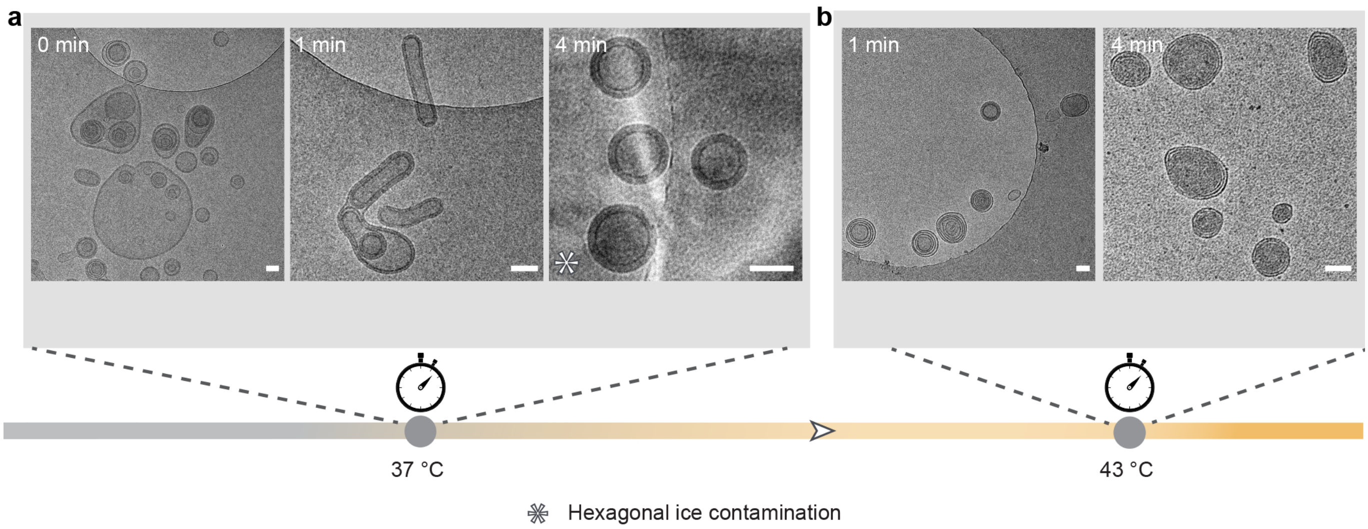

Figure S12. Additional snapshots of cryo-TEM images of self-assemblies: (a) 0, 1, and 4 min at 37 °C and (b) 1 and 4 min at 43 °C. Scale bars are 100 nm.

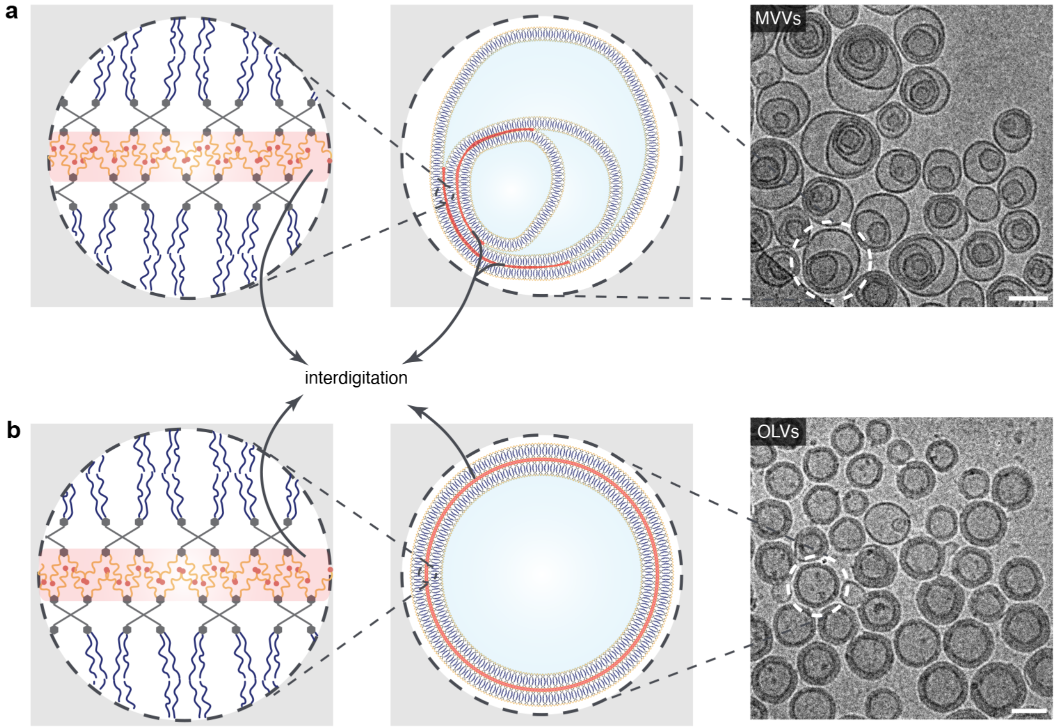

Figure S13. Interdigitation of OEG corona in MVVs and OLVs. Schematic representation of interdigitation of OEG corona of (a) MVVs and (b) OLVs. The interdigitation and noninterdigitation areas of OEG chains are highlighted with red and blue shadowed areas, respectively. The attachment of inner vesicle to the outer bilayer results in nonconcentric feature of MVVs. The complete interdigitation between inner and outer bilayer results in OLVs.

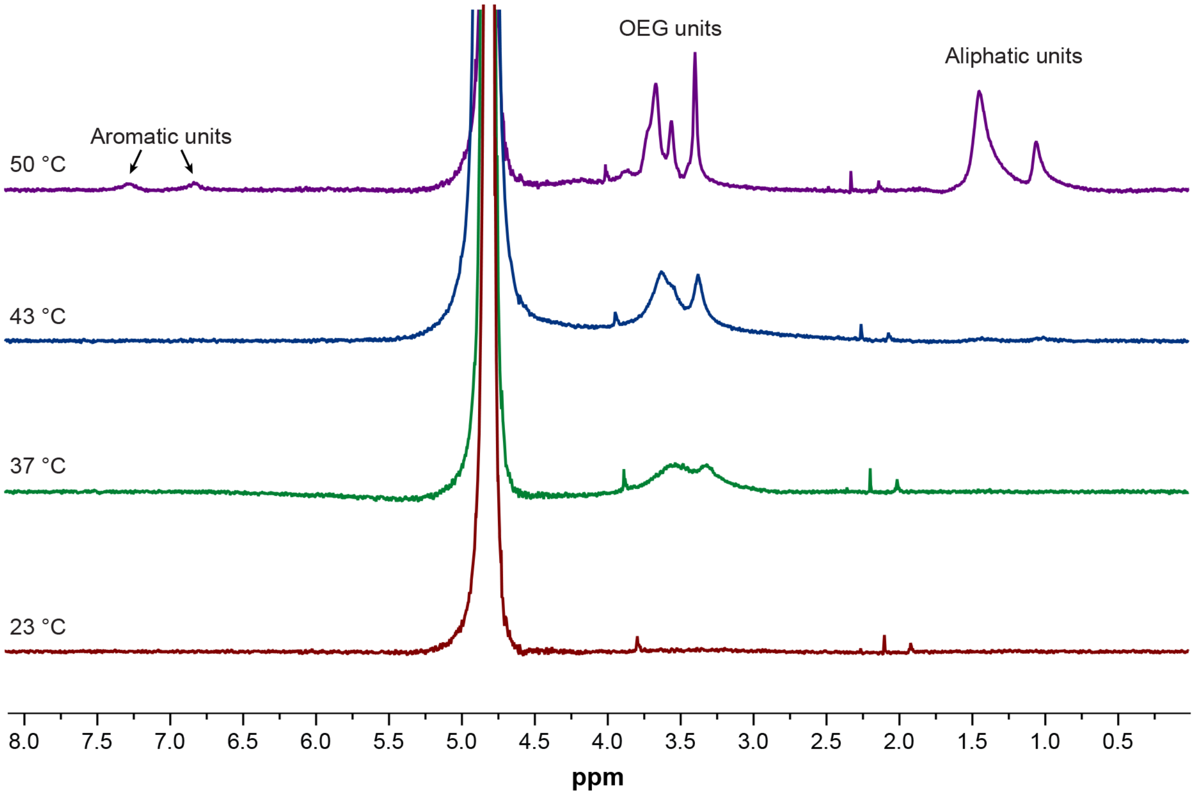

Figure S14. Variable-temperature solution-state ^{1}H NMR measurement. ^{1}H NMR spectra of self-assemblies in D_2O as a function of temperature. The solution was equilibrated for 10 min at each temperature before measurements.

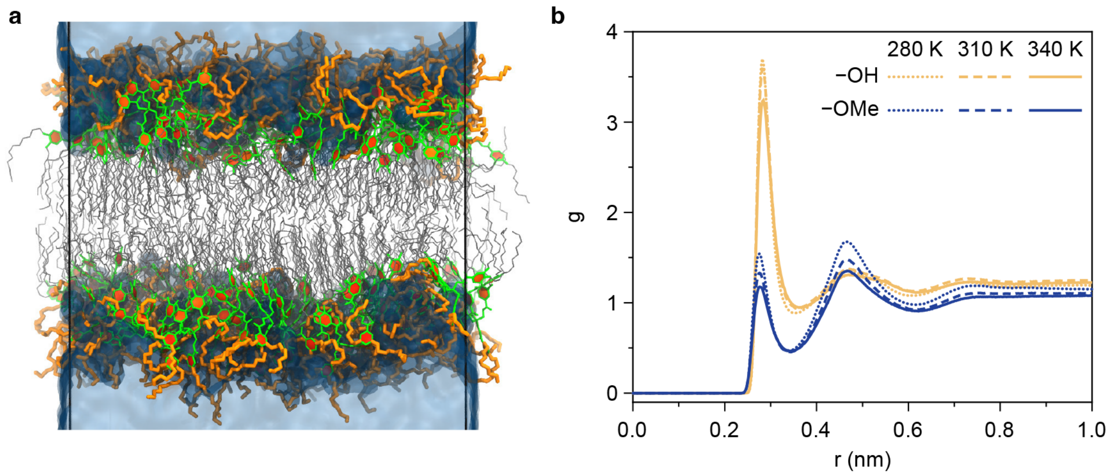

Figure S15. Molecular dynamics (MD) simulations of Janus dendrimers. (a) Snapshot of the MD simulation of Janus dendrimers with −OH end groups at 310 K. The orange, gray, and green colors represent OEGs, acyl tail chains, and other parts of the dendrimer, respectively. The blue transparent regions indicate water molecules. (b) Radial distribution functions of the oxygens of water molecules surrounding the oxygens of the end groups (i.e. −OMe and −OH) of dendrimers at 340, 310, and 280 K, respectively.

Table S1. Average number of water molecules within the first coordination sphere of oxygens of the two types of dendrimers. Each value is the integral of the corresponding RDF up to 0.35 nm.

Dendrimer	340 K	310 K	280 K
−OMe end-group dendrimer	0.78	0.82	0.96
−OH end-group dendrimer	1.96	2.04	2.19

A fully hydrated bilayer for each type of the dendrimer molecule (i.e. with −OMe and −OH end groups) was prepared using molecular dynamics (MD) method. **Figure S15a** shows a snapshot of the MD simulations of dendrimers with all −OH end groups at 310 K. The hydration properties of the two types of dendrimers were characterized by the radial distribution functions (RDFs) of the oxygens of water molecules surrounding the oxygens of the end groups at 340, 310 and 280 K, respectively (**Figure S15b**). The first peak of the RDFs for the −OH end-group dendrimer was significantly higher compared to −OMe end-group dendrimer at all three temperatures. The number of water molecules within the first coordination sphere of oxygens from the two types of dendrimers was calculated by integrating the RDFs (**Table S1**). The results showed that the number of water molecules hydrating the −OH end groups was more than twice the number of water molecules hydrating the −OMe end groups at all three temperatures. Based on these findings, it is evident that the OEG of −OH end-group dendrimer exhibits a higher degree of hydrophilicity compared to the −OMe end-group dendrimer across all temperatures investigated in the study.

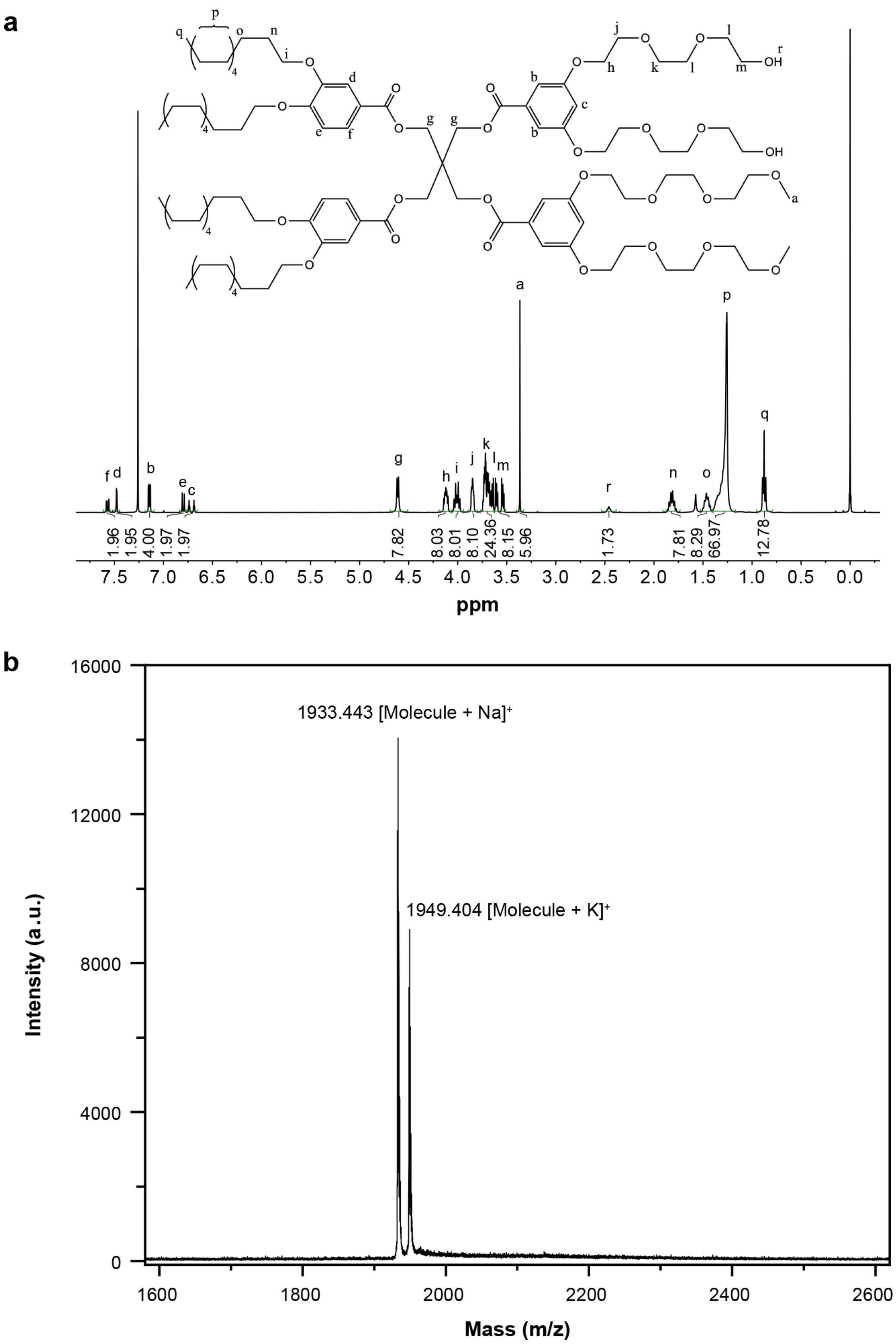

Figure S16. Characterization of (3,4)C12-PE-(3,5)-EG_3-$(OCH_3)_2$/$(OH)_2$ Janus dendrimer. (a) ^{1}H NMR in $CDCl_3$ and (b) MALDI-TOF spectra of (3,4)C12-PE-(3,5)-EG_3-$(OCH_3)_2$/$(OH)_2$.

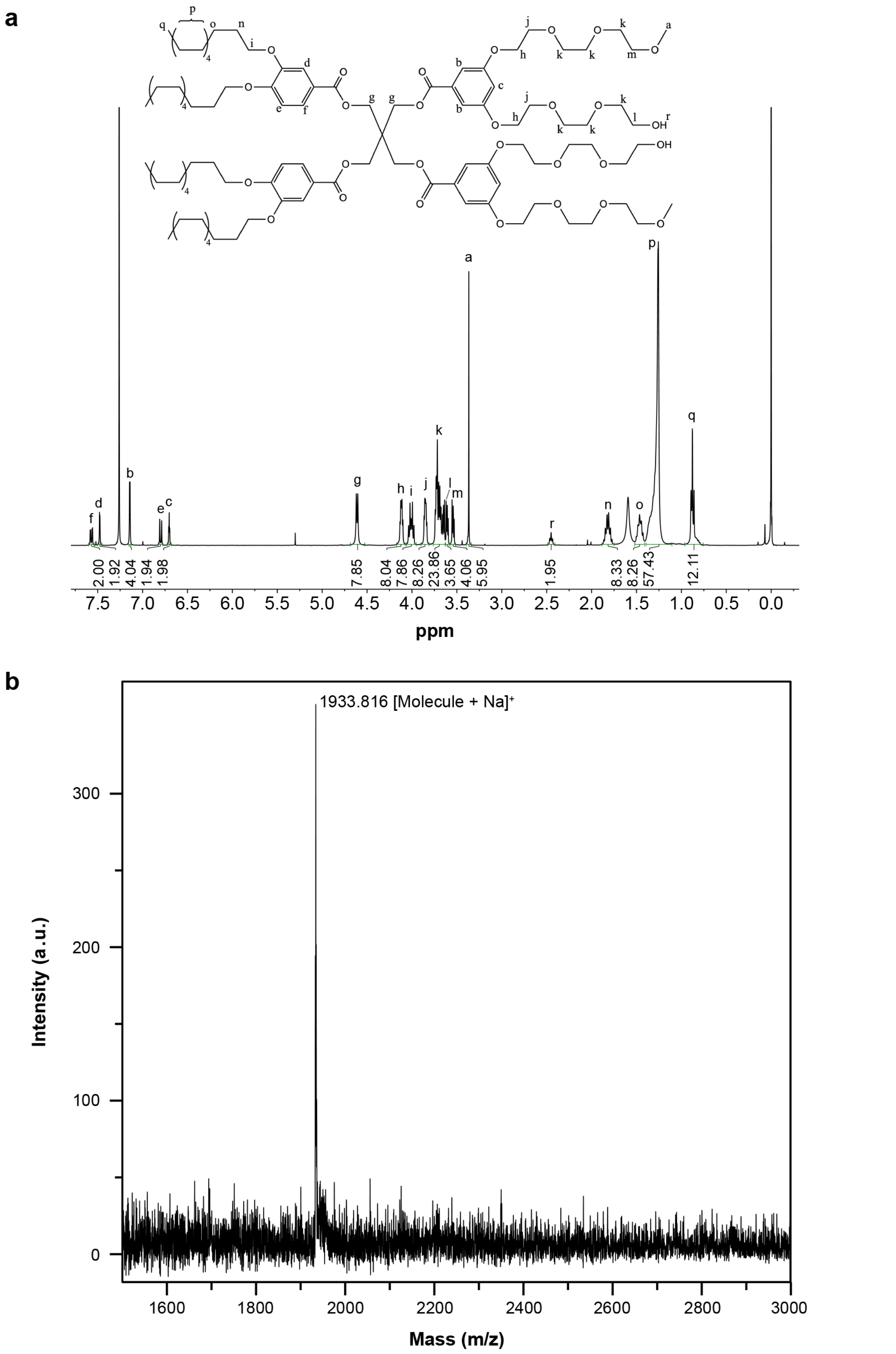

Figure S17. Characterization of (3,4)C12-PE-(3,5)-EG$_3$-(OCH$_3$/OH)$_2$ Janus dendrimer. (a) ^{1}H NMR in CDCl$_3$ and (b) MALDI-TOF spectra of (3,4)C12-PE-(3,5)-EG$_3$-(OCH$_3$/OH)$_2$.

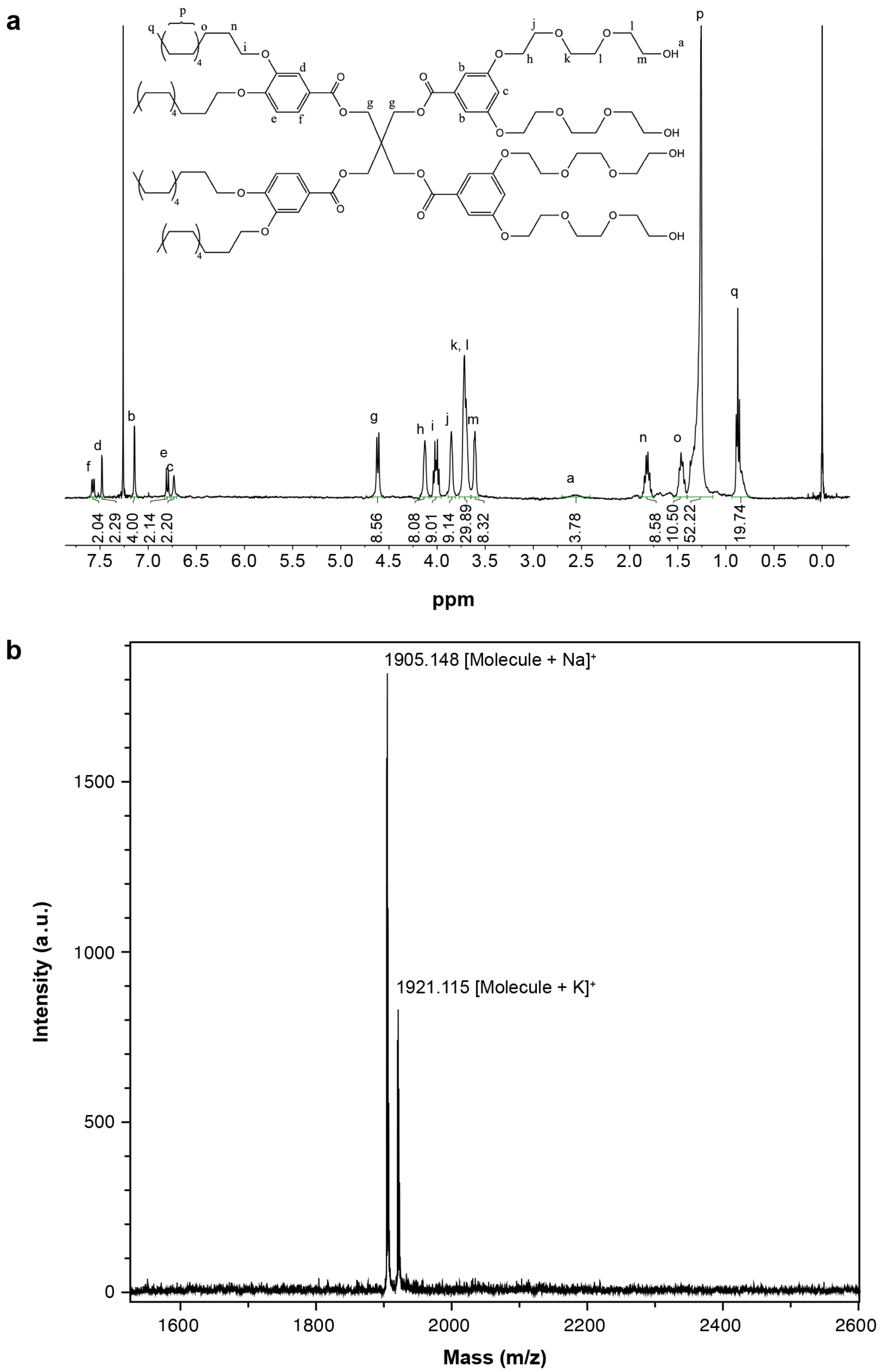

Figure S18. Characterization of (3,4)C12-PE-(3,5)-EG$_3$-(OH)$_4$ Janus dendrimer. (a) ^{1}H NMR in $CDCl_3$ and (b) MALDI-TOF spectra of (3,4)C12-PE-(3,5)-EG$_3$-(OH)$_4$.

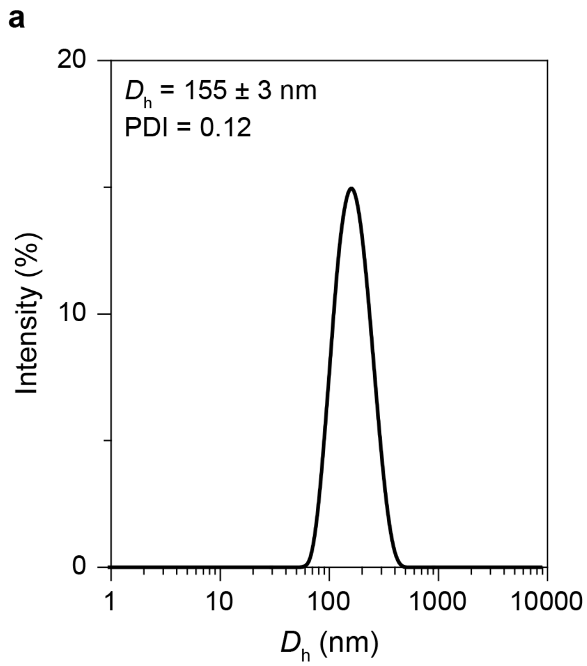

Figure S19. DLS characterization of self-assemblies of Janus dendrimers with different end groups after dialysis: (a) (3,4)12G1-PE-(3,5)-3EO-G1-$(OCH_3)_2/(OH)_2$ and (b) (3,4)12G1-PE-(3,5)-3EO-G1-$(OH)_4$.

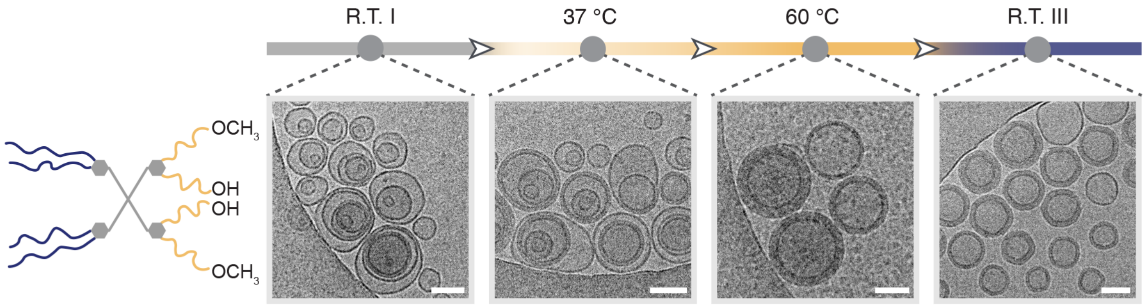

Figure S20. Assessment of the navigation of energy landscapes of assemblies from (3,4)12G1-PE-(3,5)-3EO-G1-(OCH3/OH)2. Cryo-TEM images of self-assemblies as vitrified at indicated temperatures during the first heating/cooling cycle. Scale bars are 100 nm.

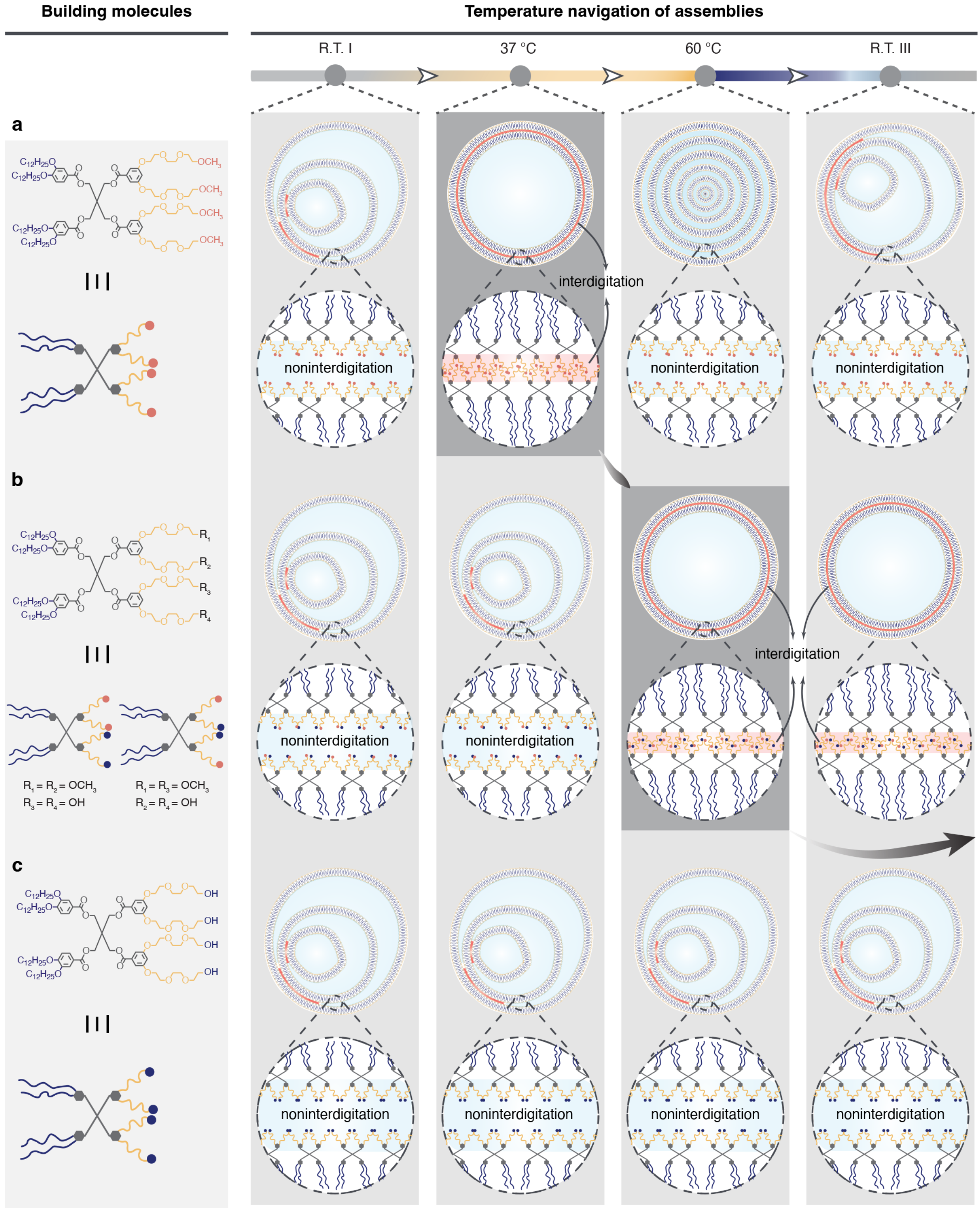

Figure S21. Energy landscapes of assembles of Janus dendrimers as navigated by the interdigitation of OEG chains. Schematic summary of the vesicles as assembled from Janus dendrimers with $–OCH_3$ end groups (a), half $–OCH_3$ and half –OH end groups (b), and –OH end groups (c) at different temperatures. The interdigitation temperatures of OEG chains of adjacent bilayers (as indicated by the dark gray arrowed lines) are shifted toward higher temperature as the molecules have more –OH end groups. Interdigitation and noninterdigitation

areas of OEG chains are highlighted with red and blue shadowed areas, respectively. Vesicles showing the full interdigitation of OEG chains at elevated temperature are highlighted with dark gray shadowed areas.

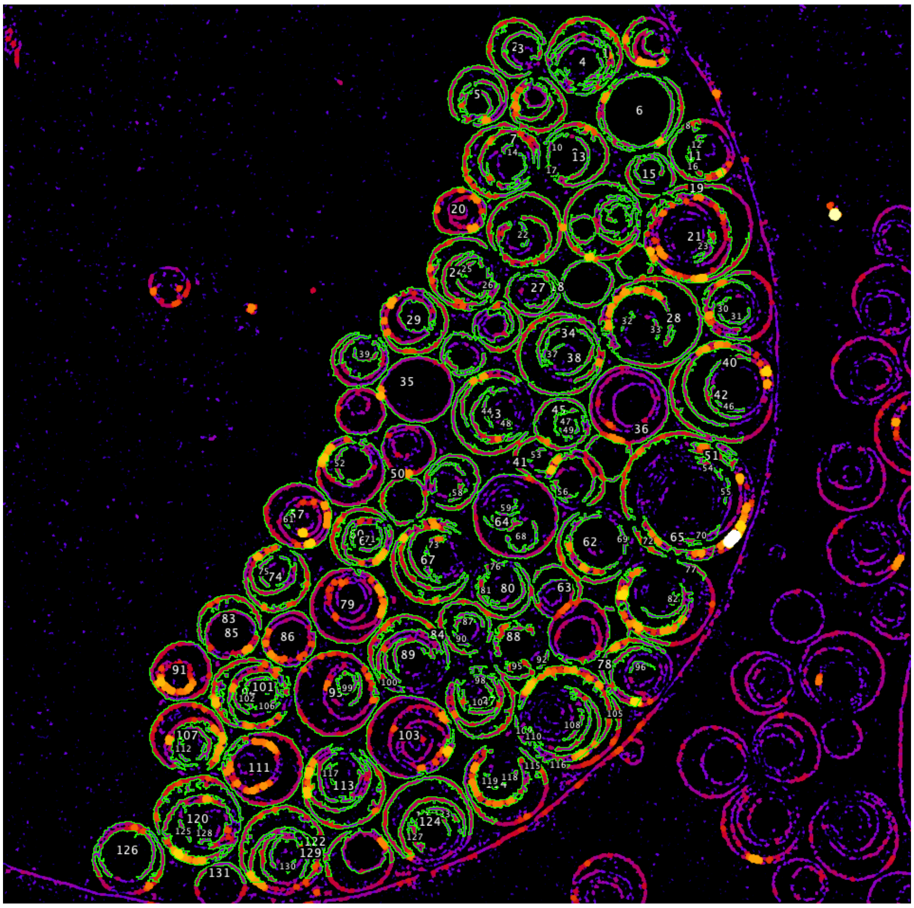

Figure S22. Quantification of membrane thickness. A typical image of selected regions of vesicular bilayers (green) after converting gray values to length, which gives the membrane thickness of each selected bilayer.

S5 | References

(1) Percec, V.; Wilson, D. A.; Leowanawat, P.; Wilson, C. J.; Hughes, A. D.; Kaucher, M. S.; Hammer, D. A.; Levine, D. H.; Kim, A. J.; Bates, F. S.; Davis, K. P.; Lodge, T. P.; Klein, M. L.; DeVane, R. H.; Aqad, E.; Rosen, B. M.; Argintaru, A. O.; Sienkowska, M. J.; Rissanen, K.; Nummelin, S.; Ropponen, J. Self-assembly of Janus dendrimers into uniform dendrimersomes and other complex architectures. *Science* **2010**, *328*, 1009–1014.

(2) Abraham, M. J.; Murtola, T.; Schulz, R.; Páll, S.; Smith, J. C.; Hess, B.; Lindahl, E. GROMACS: High performance molecular simulations through multi-level parallelism from laptops to supercomputers. *SoftwareX* **2015**, *1-2*, 19−25.

(3) Huang, J.; Rauscher, S.; Nawrocki, G.; Ran, T.; Feig, M.; de Groot, B. L.; Grubmuller, H.; MacKerell, A. D., Jr. CHARMM36m: an improved force field for folded and intrinsically disordered proteins. *Nat. Methods* **2017**, *14*, 71−73.

(4) Jorgensen, W. L.; Chandrasekhar, J.; Madura, J. D.; Impey, R. W.; Klein, M. L. Comparison of simple potential functions for simulating liquid water. *J. Chem. Phys.* **1983**, *79*, 926−935.

(5) Kim, S.; Lee, J.; Jo, S.; Brooks, C. L., 3rd; Lee, H. S.; Im, W. CHARMM-GUI ligand reader and modeler for CHARMM force field generation of small molecules. *J. Comput. Chem.* **2017**, *38*, 1879−1886.

(6) Knight, C. J.; Hub, J. S. MemGen: a general web server for the setup of lipid membrane simulation systems. *Bioinformatics* **2015**, *31*, 2897−2899.

(7) Nosé, S. A unified formulation of the constant temperature molecular dynamics methods. *J. Chem. Phys.* **1984**, *81*, 511−519.

(8) Hoover, W. G. Canonical dynamics: Equilibrium phase-space distributions. *Phys. Rev. A* **1985**, *31*, 1695−1697.

(9) Parrinello, M.; Rahman, A. Polymorphic transitions in single crystals: A new molecular dynamics method. *J. Appl. Phys.* **1981**, *52*, 7182−7190.

(10) Essmann, U.; Perera, L.; Berkowitz, M. L.; Darden, T.; Lee, H.; Pedersen, L. G. A smooth particle mesh Ewald method. *J. Chem. Phys.* **1995**, *103*, 8577−8593.

(11) Miyamoto, S.; Kollman, P. A. Settle: An analytical version of the SHAKE and RATTLE algorithm for rigid water models. *J. Comp. Chem.* **1992**, *13*, 952−962.

(12) Hess, B.; Bekker, H.; Berendsen, H. J. C.; Fraaije, J. G. E. M. LINCS: A linear constraint

solver for molecular simulations. *J. Comp. Chem.* **1997**, *18*, 1463−1472.

(13) Giuliano, C. B.; Cvjetan, N.; Ayache, J.; Walde, P. Multivesicular vesicles: preparation and applications. *ChemSystemsChem* **2021**, *3*, e2000049.

(14) Peterca, M.; Percec, V.; Leowanawat, P.; Bertin, A. Predicting the size and properties of dendrimersomes from the lamellar structure of their amphiphilic Janus dendrimers. *J. Am. Chem. Soc.* **2011**, *133*, 20507–20520.
